# Central role of Snail1 in the regulation of EMT and resistance in cancer: a target for therapeutic intervention

**DOI:** 10.1186/s13046-014-0062-0

**Published:** 2014-08-02

**Authors:** Samantha Kaufhold, Benjamin Bonavida

**Affiliations:** 1 Department of Microbiology, Immunology and Molecular Genetics, Jonsson Comprehensive Cancer Center and David Geffen School of Medicine, University of California, Los Angeles 90095, CA, USA

**Keywords:** Cancer, EMT, Metastasis, Resistance, Snail, Stem cells

## Abstract

Snail1 is the founding member of the Snail superfamily of zinc-finger transcription factors, which also includes Snail2 (Slug) and Snail3 (Smuc). The superfamily is involved in cell differentiation and survival, two processes central in cancer research. Encoded by the *SNAI1* gene located on human chromosome 20q13.2, Snail1 is composed of 264 amino acids and usually acts as a transcriptional repressor. Phosphorylation and nuclear localization of Snail1, governed by PI3K and Wnt signaling pathways crosstalk, are critical in Snail1’s regulation. Snail1 has a pivotal role in the regulation of epithelial-mesenchymal transition (EMT), the process by which epithelial cells acquire a migratory, mesenchymal phenotype, as a result of its repression of E-cadherin. Snail1-induced EMT involves the loss of E-cadherin and claudins with concomitant upregulation of vimentin and fibronectin, among other biomarkers. While essential to normal developmental processes such as gastrulation, EMT is associated with metastasis, the cancer stem cell phenotype, and the regulation of chemo and immune resistance in cancer. Snail1 expression is a common sign of poor prognosis in metastatic cancer, and tumors with elevated Snail1 expression are disproportionately difficult to eradicate by current therapeutic treatments. The significance of Snail1 as a prognostic indicator, its involvement in the regulation of EMT and metastasis, and its roles in both drug and immune resistance point out that Snail1 is an attractive target for tumor growth inhibition and a target for sensitization to cytotoxic drugs.

## Introduction

The Snail superfamily of transcription factors includes Snail1, Slug, and Scratch proteins, all of which share a SNAG domain and at least four functional zinc fingers [1]. Snail1 has four zinc fingers, located from amino acids 154 to 259, whereas Scratch and Slug each have five [2],[3]. The comparison of these zinc-finger sequences has further subdivided the superfamily into Snail and Scratch families, with Slug acting as a subfamily within the Snail grouping. The Snail superfamily has been implicated in various processes relating to cell differentiation and survival [1].

**Figure 1:**
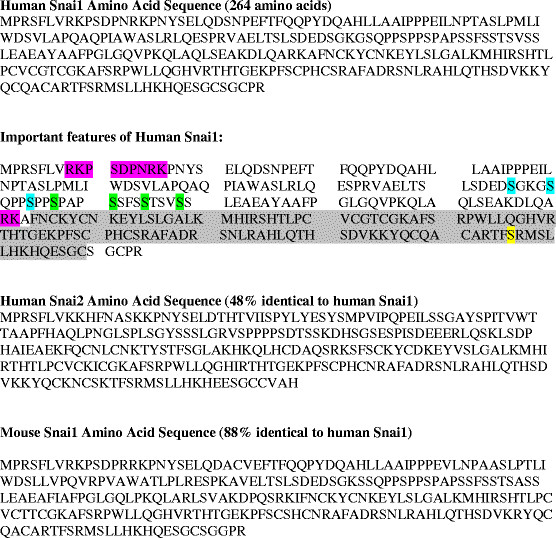
**Amino acid sequences:human and mouse.** This figure provides the human Snail1 amino acid sequence. The second representation of the sequence has important features such as phosphorylation sites and zinc fingers highlighted in various colors. 1) Purple indicates nuclear localization signals 2) blue is motif 1 for GSK-3β-mediated phosphorylation 3) green is motif 2 for GSK-3β-mediated phosphorylation 4) yellow is the PAK1 phosphorylation site and 5) gray denotes the zinc-finger region. In addition, human Snail2 (Slug) and mouse Snail1 amino acid sequences are shown for comparison to the human Snail1 sequence. Human Slug is 48% identical to human Snail1, and mouse Snail1 is 88% identical to human Snail1. The sequence alignments were run through BLAST [9].

First characterized in *Drosophila melanogaster* in 1984, Snail1 also has well-documented homologs in *Xenopus, C. elegans*, mice, chicks, and humans [4],[5]. In humans, Snail1 is expressed in the kidney, thyroid, adrenal gland, lungs, placenta, lymph nodes, heart, brain, liver, and skeletal muscle tissues [6],[7]. Snail1 is a C_2_H_2_ zinc-finger protein composed of 264 amino acids, with a molecular weight of 29.1 kDa [7] (Figure 1). The *SNAI1* gene, which is 2.0 kb and contains 3 exons, has been mapped to chromosome 20q.13.2 between markers D20S886 and D20S109 [7]. A Snail1 retrogene (*SNAI1P*) exists on human chromosome 2 [8].

Epithelial-to-mesenchymal transition (EMT) is the process by which epithelial cells lose their apical polarity and adopt a mesenchymal phenotype, thereby, increasing migratory properties, invasiveness and apoptotic resistance. The expression of mesenchymal markers, like vimentin and fibronectin, replaces that of the usual epithelial markers, including E-cadherin, cytokeratins and Mucin-1 [10]. EMT is fundamental to both normal developmental processes and metastatic cancer. The induction of epithelial-to-mesenchymal transition (EMT) is Snail1’s most studied function, as this process is crucial for the formation of the mesoderm and the neural crest [1]. Snail1 knockout in mice is lethal because gastrulation does not occur [11]. The primary mechanism of Snail1-induced EMT is the repression of E-cadherin, which causes reduced cell adhesion and promotes migratory capacity [12]. The further elucidation of Snail1’s role in EMT provides a critical insight into the development of metastatic cancer. In addition, Snail1 has been recently implicated in the regulation of drug/immune resistance and the cancer stem cell (CSC) phenotype [13]–[16].

## Regulation of Snail1 expression

### Transcriptional regulation

The Notch intracellular domain, LOXL2, NF-κB, HIF-1α, IKKα, SMAD, HMGA2, Egr-1, PARP-1, STAT3, MTA3, and Gli1 all interact directly with the Snail1 promoter to regulate Snail1 at the transcriptional level [17]–[29]. Hypoxic stress, caused by insufficient oxygen, prompts a transcriptional response mediated by hypoxia-inducible factors (HIFs) [17]. Notch increases HIF-1α recruitment to the LOX promoter, and LOXL2 oxidizes K98 and/or K127 on the Snail1 promoter, leading to a conformational change in shape [18]. Under hypoxic conditions, HIF-1α binds to HRE2, contained within -750 to -643 bp of the Snail1 promoter, and increases Snail1 transcription. Knockdown of HIF-1α results in the repression of both Snail1 and EMT [19]. NF-κB also binds to the Snail1 promoter, between -194 and -78 bp, and increases its transcription [20]. SMAD2 and IKKα bind concurrently to the Snail1 promoter between -631 and -506 bp, resulting in Snail1’s upregulation [21]. HMGA2 cooperates in this complex as well, as the binding of HMGA2 to the Snail1 promoter increases SMAD binding [22].

In addition, ILK promotes PARP-1 binding, and STAT3 binds as a final result of an IL-6/JAK/STAT pathway [23],[24]. In mice, a pathway beginning with HB-EGF and progressing through the MEK/ERK pathway has also induced STAT3 binding to the Snail1 promoter [25]. Gli1 and Snail1 interact through a positive feedback loop: Shh and Wnt crosstalk results in the upregulation of both [26]. MTA3, a subunit of the Mi-2/NuRD complex, transcriptionally represses Snail1 in an ER-dependent manner. Snail1, in turn, binds to the ER promoter to complete the negative feedback loop [27],[28]. In a similar fashion, Egr-1 and Snail1 relate via a negative feedback loop. Egr-1, another zinc-finger transcription factor, binds to the Snail1 promoter at four sites between -450 and -50 bp. This process necessitates the presence of HGF and is mediated by the MAPK pathway, and it ultimately results in Snail1 upregulation. Snail1, in turn, represses Egr-1 [29].

**Figure 2:**
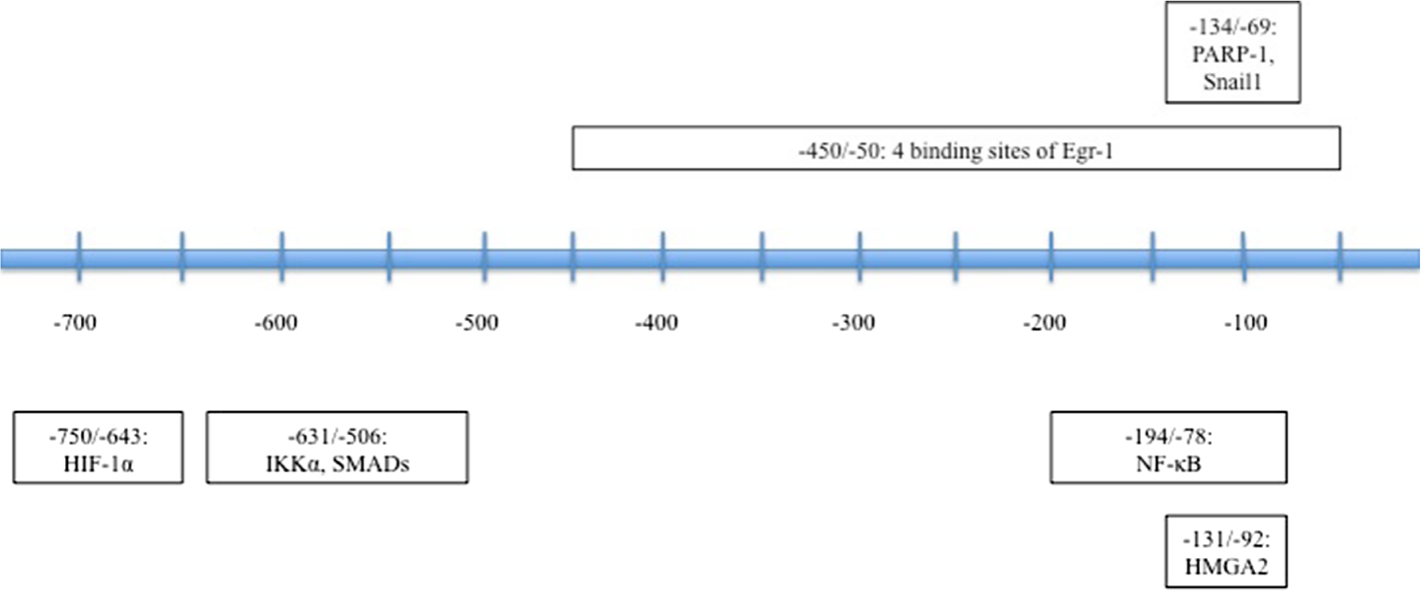
**Regulation at the Snail1 promoter.** This figure depicts the regulatory interactions at the human Snail1 promoter. The central line represents the base-paired sequence, with -750 to -1 bp shown. The relative locations of interactions with various transcription factors are then spatially compared using blocks to represent each regulator’s binding site. Each block, with the base pairs involved denoted at the top, shows where that particular protein binds the Snail1 promoter.

YY1 and Snail1 itself are two special instances of transcriptional Snail1 regulation. YY1 binds to the 3’ enhancer, rather than the promoter, and knockdown of YY1 has been shown to decrease Snail1 expression [30]. Furthermore, Snail1 is capable of binding to its own promoter and upregulating itself [31]. Snail1 binds to the E box region within the Snail ILK Responsive Element (SIRE); PARP-1 also binds to the SIRE, which is located between -134 and -69 bp, when induced by ILK [23] (Figure 2).

Experiments conducted to elucidate the relationship between p53, a tumor suppressor protein, and Snail1 have shown that p53 acts via miR-34a, -34b, and -34c to repress Snail1 at a 3’ untranslated region (UTR). Consequently, when p53 is repressed, the repression of Snail1 is lifted, and the expression of Snail1 rises [32].

### Translational regulation

**Figure 3:**
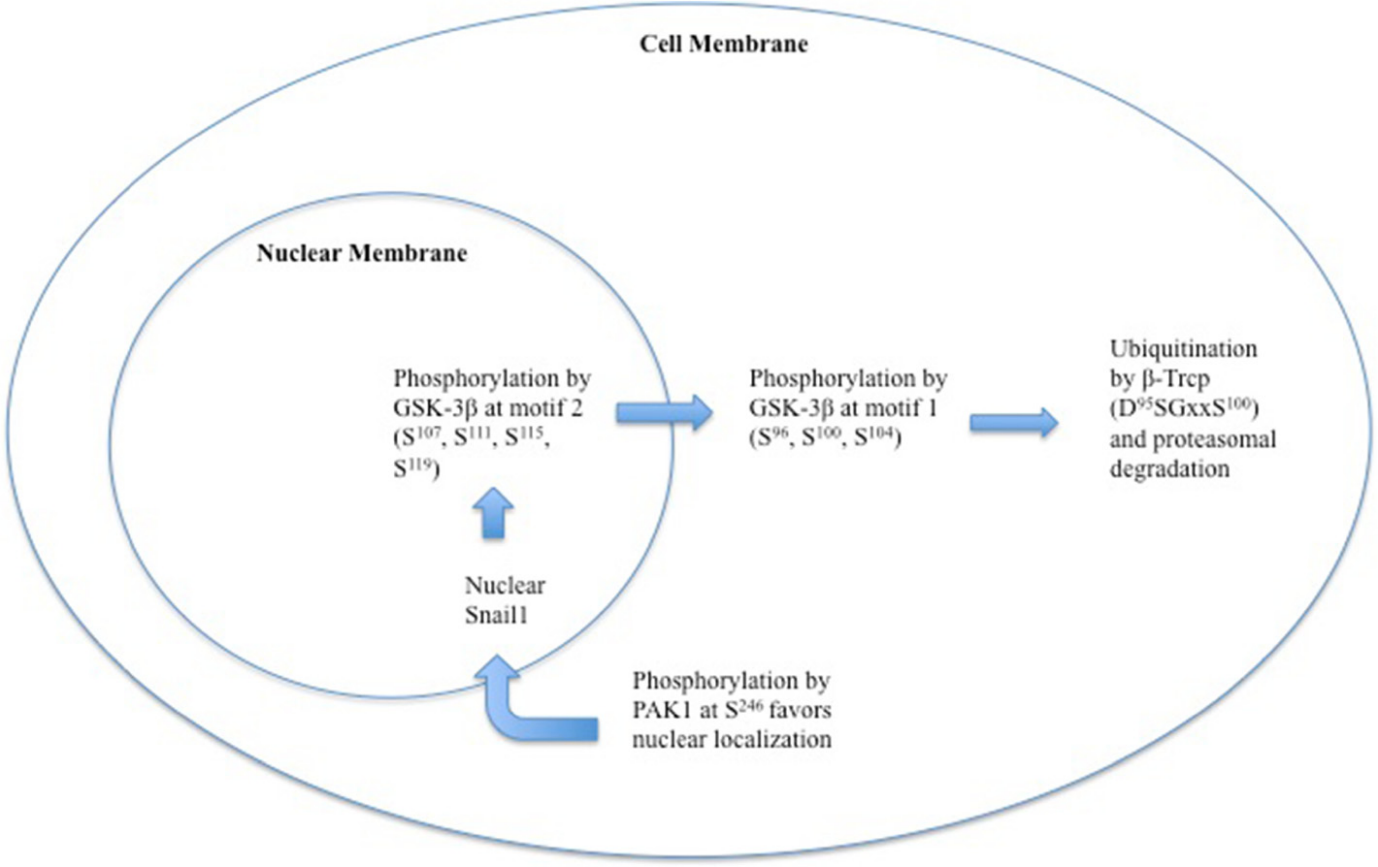
**Snail1 stability and localization.** This figure shows the effects of GSK-3β and PAK1-mediated phosphorylation on Snail1 stability and subcellular localization. The outer circle represents the cell membrane, and the inner circle represents the nucleus. Nuclear Snail1 is phosphorylated by GSK-3β at motif 2 and is consequently exported from the nucleus. If Snail1 remains in the cytoplasm, it is ultimately ubiquitinated and degraded. By contrast, phosphorylation by PAK1 favors the nuclear localization of Snail1, which increases its stability.

Two instances of phosphorylation are crucial to Snail1’s post-transcriptional regulation. GSK-3β phosphorylates Snail1 at two consensus motifs in serine-rich regions. The first phosphorylation, at motif 2 (S^107^, S^111^, S^115^, S^119^), results in Snail1’s being exported to the cytoplasm. The second instance of phosphorylation (S^96^, S^100^, S^104^) leads to its ubiquitination by β-Trcp, which recognizes the destruction motif D^95^SGxxS^100^ and ubiquitinates Lys98, 137, and 146. Consequential proteasomal degradation follows [33],[34]. In conditions that prevent GSK-3β from phosphorylating Snail1, the F-box E3 ubiquitin ligase FBXL14 appears to cause proteasomal degradation by ubiquitinating the same lysine residues as β-Trcp [35]. P21-activated kinase 1 (PAK1) also phosphorylates Snail1 at S^246^[36]. Phosphorylation determines Snail1’s subcellular location, as GSK-3β -mediated phosphorylation induces Snail1’s export to the cytoplasm through exportins such as chromosome region maintenance 1 (CRM1) [33],[37]. By contrast, PAK1 phosphorylation promotes Snail1’s presence in the nucleus and, therefore, increases its activity [36]. In the cytoplasm, Snail1 is quickly degraded; it has a half-life of only twenty-five minutes [33]. To protect from this degradation, Snail1 has nuclear localization signals (NLS): one monopartite from amino acids 151-152 and one bipartite overlapping the SNAG domain between amino acids 8 and 16 [38]. These signals are responsible for the nuclear transport of Snail1, which in turn is required for proper expression. β-catenin, Lef-1, and IκB employ similar systems [38] (Figure 3, Table 1).

**Table 1:** Regulation of Snail1 expression

**Direct regulators**	**Interaction location**	**Upstream pathway(s)**	**Reference(s)**
LOXL2/3	SNAG domain; K98 and K127	Notch/Lox	[17]
NF-κB	Promoter: -194 to -78 bp	TNFα, RANKL, PI3K/Akt	[20],[43],[44]
HIF-1α	Promoter: -750 to -643 bp	Hypoxic conditions	[19]
SMADs	Promoter: -631 to -506 bp	TGF-β1, Ras	[45],[46]
IKKα	Promoter: -631 to -506 bp (concurrent with SMADs)	TGF-β1, Ras, PI3K/Akt	[21],[44],[46]
HMGA2	Promoter: 2 regions within -131 to -92 bp	TGF-β1	[22]
YY1	3’ Enhancer	NF-κB	[30]
Egr-1	Promoter: 4 sites between -450 and -50 bp	HGF, MAPK	[29]
PARP-1	Promoter: SIRE	ILK	[23]
Gli1	There are 4 candidate GLI binding sites (consensus sequence for binding: 5'-GACCACCCA-3')	Shh, Wnt	[26]
STAT3	Promoter	IL-6/JAK, HB-EGF/EGFR/MEK/ERK (mice)	[24],[25]
MTA3	Promoter	ER	[27],[28]
PAK1	S^246^		[36]
GSK-3β	Motif 1 (S^96^, S^100^, S^104^) and Motif 2 (S^107^, S^111^, S^115^, S^119^)	Wnt, PI3K/Akt, FGF	[33],[34]
Snail1	Promoter: E box within SIRE	Binds to own promoter	[31]

TNFα, NF-κB, FGF, Wnt, and microRNA signals also influence the regulation of GSK-3β-mediated phosphorylation. The TNFα/NF-κB pathway induces CSN2, which protects Snail1 from degradation by interfering with the binding of GSK-3β and β-Trcp. Thus, Snail1 is neither phosphorylated nor ubiquitylated [39]. FGF operates through the PI3K/Akt pathway to downregulate GSK-3β, and receptor tyrosine kinase induces EGF suppression of GSK-3β [34],[40]. Wnt can also suppress GSK-3β and, thus, the phosphorylation of Snail1 [41]. Additionally, miR-148a causes the phosphorylation of AKT and GSK-3β, which results in less Snail1 localized in the nucleus. This, in turn, inhibited EMT in hepatocellular carcinoma [42].

Phosphorylation of upstream targets also influences the regulation of Snail1. For example, RANKL, in association with IκB, activates the NF-κB p65 subunit, and Akt influences the nuclear localization of NF-κB through its phosphorylation of IKKα and IκB in turn [43],[44]. TGF-β1 induces the phosphorylation of SMAD2 and SMAD3, which is necessary for their binding to Snail1 and the consequential upregulation of Snail1’s activities [45]. However, the cooperation of Ras signals is required for this pathway, since TGF- β1-mediated induction of Snail1 ceases with the silencing of Ras [46].

Other mechanisms of regulation contribute to the expression levels of Snail1, too. The small C-terminal domain phosphatase (SCP) induces dephosphorylation of both GSK-3β and the affected Snail1 motifs, thereby stabilizing Snail1 [47]. Additionally, histone deacetylase inhibitors promote the acetylation, likely of lysines, and increase Snail1’s nuclear localization by inhibiting ubiquitination [48].

## Snail1’s targets

The variety of targets regulated by Snail1, detailed below, show that Snail1’s EMT program is driven by multiple mechanisms (Table 2). While it directly represses epithelial markers like E-cadherin and claudins, Snail1 also upregulates markers of the mesenchymal phenotype, including vimentin and fibronectin. Frequently, the expression levels of Snail1’s targets serve as prognostic indicators. For example, decreased E-cadherin expression correlates with lower patient survival rates while overexpression of MMPs associates with invasiveness. In addition to replacing epithelial with mesenchymal markers, Snail1 upregulates co-repressors, as in the case of ZEB-1, to complete its EMT program.

**Table 2:** Gene targets regulated by Snail1

**Target**	**Target significance**	**Snail’s effect**	**Reference(s)**
E-cadherin	Epithelial marker, adherens junctions	Repression	[56],[57],[59]–[61]
RKIP	Tumor suppressor	Repression	[68]
PTEN	Tumor suppressor	Repression	[70]
Occludin	Epithelial marker, tight junctions	Repression	[13],[75]
Claudins	Epithelial markers, tight junctions	Repression	[75]
Mucin-1	Epithelial marker	Repression	[83]
ZEB-1	Assists in induction of EMT	Upregulation	[83]
Vimentin	Mesenchymal marker	Upregulation	[54]
Fibronectin	Mesenchymal marker	Upregulation	[54]
Cytokeratin 18	Epithelial marker	Repression	[75],[83]
MMP-2/MMP-9	Mesenchymal markers	Upregulation	[113],[118]
LEF-1	Mesenchymal marker, assists in induction of EMT	Upregulation	[83],[125]

### E-cadherin

E-cadherin is a transmembrane glycoprotein responsible for calcium-dependent cell-to-cell adhesion [49]. E-cadherin is a type I cadherin encoded by the gene *CDH1*, which is located on human chromosome 16q22.1 [50]. The founding member of the cadherin superfamily, E-cadherin plays a pivotal role in cadherin-catenin-cytoskeleton complexes, and it grants anti-invasive and anti-migratory properties to epithelial cells [51]. E-cadherin expression naturally decreases during gastrulation in order to properly form the mesoderm, and its expression increases once more for kidney organogenesis [52],[53]. The *CDH1* promoter contains multiple E-boxes, and Snail1, Slug, ZEB1, ZEB2, and Twist, among others, have been shown to directly repress E-cadherin [54]. Total E-cadherin knockout in mice resulted in immediate death at implantation [55]. Decreases in E-cadherin expression correlate with epithelial-mesenchymal transition, metastasis, and lower patient survival rates [10].

Four Snail1 complexes have been identified as mechanisms of E-cadherin repression. (1) Snail1 interacts with G9a, which concurrently recruits DNA methyltransferases (DNMTs) to the E-cadherin promoter. Snail1’s zinc fingers are thought to interact with the G9a ankyrin repeats, SET domain, or both. The complex has been shown to increase H3K9me2 and decrease H3K9 acetylation [56]. (2) The Snail1-Ajuba-PRMT5 complex promotes the methylation of H4R3. This, too, operates at the E-cadherin promoter [57]. The demethylation of H3K4 by Co-REST, CtBP, and HDAC complexes also factors into the last two mechanisms [58]. (3) Snail1 works in conjunction with Sin3A and HDAC1/2 to deacetylate H3 and H4, which suppress E-cadherin [59]. (4) In perhaps the most elucidated case, the Snail1 SNAG domain interacts with the LSD1 AO domain to form a Snail1-LSD1-CoREST complex. Snail1 residues Pro2, Arg3, Ser4, Phe5, Arg8, and Lys9 have been shown to be particularly crucial to this union, since mutants could not interact with LSD1. Likewise, LSD1 requires functional Asp375 and Glu379, Glu553, Glu555 and Glu556 to cooperate with Snail1. LSD1 inhibitors, histone H3, and SNAG peptides also hamper the activity of the complex. The formation of the Snail1-LSD1-CoREST complex results in the demethylation of H3K4me2 and consequential suppression of E-cadherin, while also increasing the stability of each of the components of the complex [60]. In a proposed second step to this mechanism, Snail1 recruits Suv39H1 to the E-cadherin promoter. Similar to prior cases, the Snail1 SNAG domain interacts with the Suv39H1 SET domain to suppress E-cadherin. Knockdown of Suv39H1 restored E-cadherin expression by inhibiting H3K9me3 [61].

### RKIP

Raf kinase inhibitor protein (RKIP), a member of the phosphatidylethanolamine-binding protein (PEBP) group, suppresses metastasis by inhibiting the Raf-MEK-ERK and NF-κB pathways [62]–[65]. In prostate, breast, and colorectal cancers, among others, RKIP expression is downregulated [64],[66]. Furthermore, elevated RKIP expression is a positive prognostic indicator for survival [66],[67]. Expression levels of RKIP correlate with those of E-cadherin, another Snail1 target, as they are both repressed by means of the E-boxes in their promoters [68].

### PTEN

Phosphatase and tensin homolog deleted in chromosome 10 (PTEN) dephosphorylates phosphoinositide-3,4,5-triphosphate (PIP3) and, thus, inhibits the PI3K pathway [69]. In this way, PTEN functions as a tumor suppressor. Snail1 binds to the *PTEN* promoter, which contains two E-boxes, and represses PTEN [70]. The specificity of this interaction is emphasized by the fact that neither Slug nor ZEB1 expression significantly alters PTEN levels [70]. Snail1’s association with the *PTEN* promoter inhibits the binding of p53, which activates PTEN during apoptosis, and it consequently increases resistance to gamma radiation-induced apoptosis [70],[71]. A positive feedback loop has been established around this interaction as well, since the repression of PTEN increases the expression of Akt [72]. Akt, operating through NF-κB, increases the expression of Snail1 [44]. Through this pathway, Snail1 may contribute to raising its own expression levels [70].

### Occludin

Occludin, an integral membrane protein crucial to the integrity of tight junctions, was first identified in 1993. The transmembrane protein has four hydrophobic domains within its 522 amino acid sequence and a molecular weight of 65 kDa [73],[74]. Though it is considered similar to connexins in gap junctions, occludin is found exclusively at tight junctions in epithelial and endothelial cells [73]. Snail1 functions as a transcriptional repressor of occludin, just as it does E-cadherin in adherens junctions. By binding to the E-box in the occludin promoter sequence, Snail1 can completely repress the promoter activity [75]. Immunoblot analysis and immunocytochemistry confirm the considerable reduction of occludin expression in the presence of Snail1 [13]. This repression, along with that of E-cadherin and claudins, is critical to the loss of cell-to-cell adhesion observed in EMT.

### Claudins

The claudin family contains more than twenty members, all of which are integral proteins spanning the membrane four times. Family members range from 20-27 kDa, but they all share PDZ binding motifs, which allow them to interact with ZO-1, ZO-2, and MUPP-1, among others [76]. Claudins are components of tight junctions, and claudin-1 binds with occludin [76],[77]. The expression of claudins is frequently low or nonexistent in breast cancer cell lines, and it shares an inverse relationship with Snail1 expression levels in invasive breast tumors [77].

Specifically, claudin-1, -3, -4, and -7 are all susceptible to repression by Snail1. The promoter sequence of each of these proteins contains multiple E-box binding motifs: claudin-1 has two E-boxes, claudin-3 has six, claudin-4 has 8, and claudin-7 has eight. As such, Snail1 can completely inhibit their transcription [75]. The destruction of tight junctions that accompanies the repression of claudins and occludin leads to epithelial cells’ loss of apical polarity and increases proliferation [78]. This mechanism helps drive Snail1-induced EMT.

### Mucin-1

Mucin-1, a transmembrane glycoprotein encoded by *MUC1*, is an epithelial marker expressed at the apical surface of epithelial cells in the reproductive tract, digestive tract, lungs, kidney, liver, eyes, and other tissues [79]–[81]. Additionally, it is expressed in hematopoietic and T cells [80]. Mucin-1’s functions include lubrication and protection from pathogens, and its association with β-catenin has implicated Mucin-1 in cell signaling [80]. O-linked glycosylation affects the protein significantly, as the core protein ranges from 120-225 kDa and the glycosylated form can reach up to 500 kDa [82]. In epithelial tumors, Mucin-1 is upregulated, and disparities in splice variants and glycosylation become apparent [79],[80]. Splice variants differ greatly—the protein can vary from 4-7 kb [82]. Perhaps most importantly, Mucin-1 also loses its apical restriction in malignant cases [80].

The 2872 bp promoter facilitates much of Mucin-1’s regulation, and it notably includes five sites for YY1 binding [79]. Snail1 interacts with the two E-boxes that begin -84 bp from the start of transcription. Like E-cadherin, Mucin-1 is an epithelial marker repressed by Snail1 during the induction of EMT [83].

### ZEB-1

ZEB-1, like Snail1, is a zinc-finger transcription factor that assists in the induction of EMT. Using E-boxes and co-repressors such as CtBP and BRG1, ZEB-1 represses E-cadherin and Mucin-1 [83],[84]. However, ZEB-1 is at least ten times less potent a repressor of both E-cadherin and Mucin-1 than Snail1 [83]. Interference with the interaction between ZEB-1 and BRG1 results in the upregulation of E-cadherin and simultaneous downregulation of vimentin, so an abundance of functional ZEB-1 is associated with a mesenchymal phenotype [84]. In contrast to the lethal effects of Snail1 knockout, ZEB-1 knockout does not prevent development to term and, thus, is not as critical for gastrulation [83].

The presence of Snail1 increases both RNA and protein levels of ZEB-1 during EMT. Snail1 expression in MDCK clones causes a 2.5-fold increase in ZEB-1 promoter activity compared to control cells. The abilities of Snail1 and ZEB-1 to repress E-cadherin are additive, and the two transcription factors work together to achieve a complete EMT [83].

### Vimentin

Vimentin is 57 kDa intermediate filament generally restricted to mesenchymal cells [85]. Vimentin regulation is a complex interplay of epigenetic and post-translational modifications in addition to transcriptional regulation. Of note, the human vimentin promoter contains an NF-κB binding site as well as a TGF-β1 response element [86],[87]. Akt1 protects vimentin from caspase proteolysis via phosphorylation of Ser39 [88]. During EMT, epithelial cells, which normally express keratin intermediate filaments, begin to express vimentin. Overexpression of vimentin is evident in breast and prostate cancers, among many other types, and overexpression generally correlates with invasiveness, migration, and poor prognosis [89]–[91]. Snail1 upregulates vimentin during EMT [54].

### Fibronectin

Fibronectin is a glycoprotein involved in cell adhesion, differentiation, and migration [92],[93]. A dimer with two 250 kDa components, fibronectin is greatly affected by splicing, and at least twenty variants of the human form have been identified [94]. Fibronectin interacts with many integrins in addition to heparin, collagen, and fibrin [95]–[99]. Inactivation of fibronectin is lethal in mice [100]. Snail1 upregulates fibronectin, a mesenchymal marker indicative of EMT [54].

### Cytokeratin 18

Cytokeratins exist in two types, and each cytokeratin works with a complementary partner to form keratin filaments [101]. Cytokeratin 18 is the first type, acidic, and interacts with the basic cytokeratin 8 [101]. The cytokeratin 18 protein is encoded by the *CK18* gene, which is located on chromosome 12q13. Cytokeratin 18 is an intermediate filament protein involved in cell structure, cell signaling, and the cell cycle [101]–[104]. Cytokeratin 18 serves as an epithelial marker, and it localizes in epithelial organs, such as the kidney, liver, gastrointestinal tract, and mammary glands [105]. Snail1 represses cytokeratin 18 during the induction of EMT [83]. Unlike other targets, though, cytokeratin 18 expression is not completely subdued by Snail1’s presence [75].

### MMP 2/9

Matrix metalloproteinases (MMP) cleave extracellular matrix substrates and, thereby, alter cell-matrix adhesions [106]. MMP-2 and -9 are a subcategory within the MMP group because they specifically act on gelatin, collagen, elastin, and fibronectin [107]–[111]. The genes that encode MMP-2 and -9 both contain fibronectin type II domains and are consequently three exons longer than the other MMP genes [107]. MMP-2 is a 72 kDa protein while MMP-9 is 92 kDa, and the main difference between them is the MMP-9’s 54 amino acid hinge region [107],[112]. Additionally, MMP-2 localizes in the nucleus and MMP-9 in the cytoplasm [113]. Overexpression of MMP-2 and MMP-9 is frequently associated with invasive, metastatic tumors [114]–[117].

Snail1’s presence increases the mRNA levels of both MMP-2 and -9 [118]. One suggested interaction includes the upregulation of MMP-2 and -9 by Snail1 to trigger EMT and, then, the coordinated effort of Snail1 and Slug to sustain EMT by continually stimulating MMP-9 [113].

### LEF-1

Lymphoid enhancer-binding factor 1 (LEF-1) is a T-cell factor commonly detected in tumors [119],[120]. The transcription factor represses E-cadherin by forming complexes with β-catenin, which, like Snail1, is degraded as a result of GSK-3β-mediated phosphorylation [11],[121]–[123]. LEF-1 interacts with Snail1 via Wnt, PI3K and TGF-β1 pathways, and both Snail1 and LEF-1 are necessary for a complete EMT [124]. LEF-1 is considered a mesenchymal marker, and Snail1 induces its expression and continues to upregulate it [82],[125].

## Snail1 expression in cancer

Snail1 is expressed in many types of cancer. Snail1 overexpression usually correlates with increased migration, invasion, and metastasis. An inverse relationship with E-cadherin is expected, and Snail1 consequently corresponds with poor differentiation as well. Frequently, more advanced malignancies and poor prognosis also accompany elevated Snail1 expression (Table 3).

**Table 3:** Snail1 expression in cancers (listed in alphabetical order)

**Type of Cancer**	**Snail Expression**	**Change in expression over disease progression**	**Prognostic significance**	**Reference(s)**
Bladder carcinoma	Inverse correlation with E-cadherin [130]; low expression level (only 16% of carcinoma tissues, n = 120)—Slug and Twist appear to be more important [140]	Increased expression in node-positive vs. node-negative tumors; 3 year progression free survival rate with positive Snail expression only 15% [140]	Snail expression associated with tumor recurrence; elevated expression is significant, independent prognostic factor [140],[141]	[130],[140],[141]
Breast carcinoma	None in normal breast epithelium; in 47% of IDCs (n = 17); none in ILCs (n = 4); expression correlates with lymph node metastases; not found in cells with constitutively inactive E-cadherin; expression opposes E-cadherin [128]; Snail overexpressed; Snail/E-cadherin ratio significantly higher [129]	Snail expression in IDCs (n = 17): grade 1—none; grade 2—more than half; grade 3—most [128]	Potential marker of IDC malignancy [128]; High expression correlated with shorter effusion-free, disease-free, and overall survival; correlated with lymph node metastases and high histologic grade (n = 16) [129]	[128],[129]
Cervical carcinoma	Snail expressed in 94% of samples (n = 70) and ZEB-1 in 96%; nuclear expression of both correlates with advanced FIGO stage and lymph node metastasis; expression of Snail correlates with poor differentiation [153]	High Snail expression associated with late FIGO stage, lymph node metastasis, and poor differentiation [153]		[153]
Colorectal carcinoma	Inverse correlation with E-cadherin [130]; markedly high expression (78% of tested tumors, n = 59) of Snail; Snail-positive in older age group than Snail-negative (mean 58.9 vs. 49.8, n = 59) [138]; Snail expressed in all tested CRC cell lines (Western blot); expression increased migration and invasiveness; decreased E-cadherin; led to CSC-like phenotype and spindle morphology [139]	Increase in expression over disease progression: 15/23 stage III vs. 6/6 stage IV [138]; significantly higher rate of metastasis among Snail-expressing than Snail-negative [139]	Snail expression indicates high risk of distant metastases [138],[139]	[130],[138],[139]
Gastric carcinoma	Snail expression higher in diffuse than intestinal type [134]; inverse correlation with E-cadherin; significantly reduced E-cadherin expression; Snail expression more comparable to breast than ovarian carcinoma [129]	Overexpression associated with tumor size, depth of invasion, lymph node metastasis, shortened survival [135]	Considered independent predictor of poor prognosis [135]	[129],[134],[135]
Hepatocellular carcinoma	Inverse correlation of mRNA and protein levels with E-cadherin (E-cadherin in Hep-G_2_ while Snail in HuL-1, Changliver, HLE, and HLF cells) [130]; Snail correlates with invasiveness and metastasis, Snail overexpression in 23% of cases (n = 47) [131]	Higher Snail expression in higher grade cases (n = 12) [132]	Risk factor for early recurrence (n = 47) [131]; Snail correlated with portal vein invasion, metastasis, poorer prognosis in recurrence-free survival [132]	[130]–[132]
Melanomas	High mRNA expression in all tested melanoma cell lines but not primary melanocytes; low E-cadherin in presence of Snail [142]; inverse correlation with E-cadherin [130]; Snail confers invasive and immunosuppressive properties [143]			[130],[142],[143]
Oral squamous carcinoma	Low E-cadherin/high Snail expression cells more invasive; E-cadherin positive had cuboidal shape and E-cadherin negative cells were spindle-shaped; inverse correlation with E-cadherin [136]			[136]
Ovarian carcinoma	Less expressed than in breast carcinoma [129]; lower expression in effusions than primary tumors and solid metastases; mRNA levels not statistically different among the three; complete cytoplasmic localization in effusions [133]		High E-cadherin expression correlated with disease-free survival; MMP-2 is considered a marker of poor prognosis; Snail associated with distant metastases [129]	[129],[133]
Pancreatic carcinoma	Inverse correlation with E-cadherin [130]; significantly reduced E-cadherin expression [129]; 78% of tested tissues (n = 36, ductal adenocarcinoma) showed Snail expression; Snail higher in undifferentiated cell lines (MiaPaCa-2 and Panc-1) than differentiated (Capan-1, HPAF-2, AsPC-1) [137]			[129],[130],[137]
Prostate cancer	Significant loss of E-cadherin and syndecan 1 in high grades, along with high Snail; only nuclear localization in PC3 cell lines [151]	High Snail expression correlates with high Gleason grade, increased malignancy [151]		[151]
Synovial sarcoma	Snail mRNA found in all cases tested (n = 20), but E-cadherin mutations appear to be more important than Snail expression [144]			[144]

### Breast carcinoma

Invasive breast carcinomas, including infiltrating ductal (IDC) and infiltrating lobular carcinomas (ILC), spread to surrounding breast tissues, lymph nodes and the pleural cavity. Assigned histological grades, with three being the highest, correlate with prognosis [126]. Breast carcinomas can give rise to malignant pleural effusions, and typical survival rates at that point are a matter of months [127].

Snail1 is not present in normal breast epithelium, nor is it present in ILCs (n = 21). Of 17 patients, Snail1 was expressed in 47% of IDCs, and its expression correlated with lymph node metastases and high histologic grades [128]. E-cadherin and Snail1 expression levels are inversely related, and high expression levels of Snail1 correlate with shorter effusion-free, disease-free, and overall survival rates (n = 16) [129]. As such, Snail1 has prognostic significance as a marker of IDC malignancy [128].

### Hepatocellular carcinoma

Snail1 mRNA and protein levels are inversely correlated with E-cadherin in hepatocellular carcinoma (HCC) [130]. Snail1 overexpression, which in one study included 23% of cases (n = 47), is associated with portal vein invasion, metastasis, and poor differentiation. Furthermore, Snail1 expression correlates with a poor prognosis in recurrence-free survival and, thus, is considered a potential risk factor for early recurrence [131],[132].

### Ovarian carcinoma

Overall, Snail1 expression is lower in ovarian carcinoma than in breast carcinoma, though its expression is still associated with distant metastases [129]. Expression is higher among primary tumors and metastases than effusions, and effusions show complete cytoplasmic localization of Snail1 [133]. Snail1 represses E-cadherin and upregulates MMPs, and E-cadherin expression correlates with disease-free survival while MMP-2 is considered a marker of poor prognosis [129].

### Gastric carcinoma

E-cadherin expression is drastically reduced in gastric carcinoma, and Snail1 expression levels once again share an inverse relationship with E-cadherin expression levels [129]. Snail1 expression levels are more comparable to breast than ovarian carcinomas, and Snail1 expression is still higher in diffuse rather than intestinal varieties of gastric carcinomas [129],[134]. Elevated Snail1 expression increases cells’ capacities for migration and invasion. Overexpression correlates with tumor size, depth of invasion, and lymph node metastasis. Shortened survival rates are also directly related to Snail1 overexpression, and Snail1 is considered a predictor of poor prognosis [135].

### Oral squamous carcinoma

Oral squamous carcinoma is another case of E-cadherin/Snail1 expression inversion, and the higher the Snail1 expression, the more invasive the cancer. E-cadherin positive cells maintain their cuboidal shape while E-cadherin negative cells turn spindle-shaped. This is a typical sign of EMT, and it shows Snail1’s repression of E-cadherin [136].

### Pancreatic carcinoma

Pancreatic carcinoma tissues show significantly reduced E-cadherin levels and relatively high Snail1 expression [129]. In one study, 78% (n = 36) of ductal adenocarcinoma tissues expressed Snail1, and Snail1 expression is higher in undifferentiated cell lines than in differentiated ones [137].

### Colorectal carcinoma

Colorectal cancer (CRC) begins in gland cells that line the colon and rectum, and it is one of the most commonly newly diagnosed cancers and a leading cause of cancer-related deaths [138]. Snail1 expression is again inversely correlated to E-cadherin expression in CRC, and the expression level of Snail1 is quite high in CRC (78%, n = 59) [130],[139]. Interestingly, the mean age of the Snail1-positive group was nine years older than the Snail1-negative group in one study, with a standard deviation of 12.7 years (58.9 years vs. 49.8 years, n = 59) [139]. In another study, Snail1 expression was detected by Western blot in all tested CRC lines, and its expression increased both migratory and invasive properties. Additionally, Snail1 expression led to a stem-cell like phenotype and spindle shape, as usually accompanies the loss of E-cadherin [140]. Snail1 expression also increased with the stage of the tumors, with 15/23 stage III expressing Snail1 and 6/6 of stage IV. The significantly higher rate of metastasis associated with Snail1 expression suggests that Snail1’s presence indicates a high risk of distant metastases [139],[140].

### Bladder carcinoma

Though the expression level of Snail1 is lower in bladder carcinoma than in other types of cancer, its presence still has a significant impact on the cancer’s progression. In one study, only 16% of the 120 tested tissues expressed Snail1, indicating that Slug and Twist, whose expression levels were 63% and 44% respectively, play larger roles. However, Snail1 expression increased in node-positive compared to node-negative tumors, and Snail1’s presence lowered the three-year progression free survival rate to only 15% [141]. Since Snail1 expression is closely linked with tumor recurrence, its elevation is considered a significant prognostic factor [141],[142].

### Melanoma

In melanoma, there is increased Snail1 mRNA and low E-cadherin in the presence of Snail1 expression. By contrast, no Snail1 mRNA was detected in primary melanocytes [143]. Snail1 expression confers both invasive and immunosuppressive properties in melanoma [144].

### Synovial sarcoma

Saito et al. reported that Snail1 mRNA was found in all cases tested of synovial sarcoma (n = 20) and E-cadherin mRNA was detected by RT-PCR in 14/20 cases. This does not show the same strong inverse correlation that has come to be expected of Snail1 and E-cadherin. In this case, mutations of the *CDH1* gene, which encodes E-cadherin, seem to be more influential than the presence of Snail1 [145].

### Prostate cancer

Prostate cancer is the second most commonly diagnosed cancer in men worldwide, with estimates of over 900,000 new cases per year [146]. A Gleason grade, which describes the two most important histopathological patterns of that patient’s cancer, accompanies a diagnosis. The grade ranges from 2-10 with a higher score meaning less differentiated [147]. Significant losses of E-cadherin and syndecan 1, two proteins involved in cellular adhesion, have been observed in malignant prostate cancer [148],[149]. Both promoters contain E-boxes, so Snail1 can directly bind and repress them [150],[151]. The presence of E-boxes may explain the inverse correlation between E-cadherin/syndecan 1 and Snail1 expression levels. Poblete et al. found that high Snail1 expression correlated with a high Gleason grade and increased malignancy. Furthermore, in more malignant cell lines, like PC3, Snail1 had exclusively nuclear localization. By contrast, Snail1 had both cytoplasmic and nuclear localization in less malignant cell lines [152].

### Cervical carcinoma

Cervical cancer is one of the most common malignancies in women worldwide [138]. Chen et al. found Snail1 expressed in 94% of samples (n = 70), and the elevated expression of Snail1 correlated with late FIGO stage, lymph node metastasis, and poor differentiation [153].

## Snail1 and cancer stem cells

Snail1-induced EMT causes a stem-like phenotype, a property closely related to metastasis and resistance. Cancer stem cells (CSCs), or tumor-initiating cells, are subpopulations within tumors that possess self-renewing capabilities [154]. In breast tissue, for example, populations with a CD44^high^/CD24^low^ phenotype have a higher tumor-initiating capacity than do their CD44^low^/CD24^high^ counterparts within the same tumors [155]. CSCs are also associated with chemoresistance, relapse, and metastasis [156].

Mani et al. reported that EMT could induce stem-like properties in non-cancerous mammary epithelial cells [14]. The CD44^high^/CD24^low^ phenotype correlates with both breast CSCs and normal mammary stem cells, and both Snail1- and Twist-induced EMTs stimulated this same phenotype in nontumorigenic human mammary epithelial cells (HMLEs). These EMTs also increased the HMLEs’ mammosphere-forming ability thirty-fold, and the CD44^high^/CD24^low^ cells are able to produce more CD44^high^/CD24^low^ cells in addition to CD44^low^/CD24^high^ cells. Furthermore, these CD44^high^/CD24^low^ cells exhibited a decrease of E-cadherin expression along with elevated fibronectin, vimentin, Snail1, and Twist, as measured by RT-PCR [14]. Thus, EMT promotes self-renewal capabilities and the stem-like phenotype.

Given that Snail1 induced EMT and a stem-like phenotype in human colorectal cancer cells (as mentioned in “Colorectal Carcinoma,” above), Zhou et al. examined human pancreatic cancer cells and reached similar conclusions [15]. Epithelial BxPC-3 cells were compared with more morphologically diverse Panc-1 cells, and the comparison identified Panc-1 cells, which had higher Snail1 expression and were more poorly differentiated than BxPC-3 cells, as CSC^high^ with a larger ALDH^high^ population [15]. Stem cells’ pluripotent capabilities are maintained in part by the polycomb complex protein BMI-1 (Bmi-1), homeobox protein Nanog, sex-determining region Y-box 2 (Sox2), and octamer-binding transcription factor 4 (Oct4) [157]–[159]. Snail1 silencing resulted in a decrease in ALDH, Sox-2, Oct-4, and invasive properties. Following Snail1 knockdown, E-cadherin expression increased as vimentin and ZEB1 expressions both decreased. Without Snail1, the Panc-1 cells underwent MET and consequently lost their stem-like phenotype [15].

In a similar study of non-small cell lung cancer, Wang et al. compared ciplatin-resistant A549 cells with their A549 counterparts [16]. A549/CDDP cells showed increased expression levels of Nanog, Oct4, and Bmi-1, as detected by Western blot. RT-PCR also showed increased CD44 and Sox2. Migratory and invasive capacities were increased in A549/CDDP cells, as well. Interestingly, only Snail1 expression was elevated in A549/CDDP cells—Slug, Twist, and ZEB1 were not influential factors in this comparison. Snail1 knockdown again caused a decline in migration, invasiveness, Bmi-1 expression, Oct-4 expression, and mammosphere-forming ability. E-cadherin increased as vimentin decreased, and the cells became more responsive to cisplatin [16]. Since β-catenin had effects on the system comparable to active Snail1, an antagonist of the PI3K/Akt pathway was introduced, and this resulted in a decrease in β-catenin, Snail1, Nanog, migration, invasiveness, and mammosphere-forming ability [16]. Thus, the Akt pathway plays a crucial role in stem-like phenotype in lung cancer cells.

Poor differentiation, sphere-forming capacity, self-renewal, and typical markers such as ALDH and CD44, among other properties, characterize the stem-like phenotype [15]. Clearly, Snail1 overexpression is associated with all of these properties. After Snail1 induces EMT, cells adopt a mesenchymal morphology, become more invasive, increase migratory capacity, and express a stem-like phenotype. Knockdown of Snail1 causes the reverse process, mesenchymal-epithelial transition (MET), which prompts cells to become less invasive, migratory, and stem-like, as well as more sensitized to drugs. Thus, Snail1-induced EMT is a critical link between resistance, metastasis, and stem-like characteristics.

## Regulation of EMT, in part, by Snail1

Snail1 drives EMT primarily through the direct repression of E-cadherin [53]. Other targets that contribute to Snail1’s EMT program were detailed above (See Section “Snail1’s Targets”, Table 2). However, other transcription factors, notably, TGF-β, RANKL, Notch1, and Cox-2, Notch1 are crucial to the EMT phenotype as well.

Zhu et al. have examined the relationship between the expression of the Response Gene to Complement-32 (RGC-32) and TGF-β-mediated EMT [160]. RGC-32 is over-expressed in many cancers and correlates with the lower level of expression of E-cadherin in pancreatic cancer. Stimulation of cells with TGF-β was associated with the upregulation of RGC-32 and EMT. Noteworthy, the findings that RGC-32 mediated TGF-beta-induced EMT and cell migration was corroborated with the use of RGC-32 siRNA. The authors extrapolated that RGC-32 regulates Snail1 expression and EMT.

Snail1 is a target of NF-κB activity and its expression and role in EMT are well recognized. Since NF-κB is activated by many signals, clearly, such signals will also regulate Snail1 among other target gene products. Tsubaki et al. have reported that various solid tumors express the Receptor Activator of Nuclear Factor-κB (RANK) and it is activated by RANK-ligand resulting in the promotion of tumor cell growth, migration, metastasis, and anchorage independence in breast cancer cells [42]. In addition, they reported that RANKL induces EMT by activating NF-κB and enhances the expression of Snail1, Twist, vimentin, and N-cadherin and decreases the expression of E-cadherin. Inhibitors of NF-κB are shown to inhibit RANKL-mediated EMT, cell migration, and invasion.

Huang et al. investigated the expression level of Notch1 in lung adenocarcinoma and its relationship to metastasis [161]. They found that lung tumors express low levels of Notch1 and were associated with advanced clinical stage and lymph node metastasis. In contrast, patients with positive Notch1 expression had the prolonged progression of overall survival. Thus, Notch1 expression regulates negatively the EMT phenotype. Dysregulation of the Notch signaling pathway plays an important role in the pathogenesis of many cancers. Notch1 is one receptor of the Notch signaling pathway. Notch1 is involved in the regulation of tumor cell growth, proliferation, apoptosis, metastasis, and chemoradioresistance. Notch1 protects Snail1 from degradation by preventing GSK-3β-mediated phosphorylation via LOXL2 oxidation, as detailed above [18].

The relationship between the expression of cyclooxegnase-2 (Cox-2) and the downregulation of E-cadherin and its relationship to the EMT phenotype was reported by Fujii et al. [162]. These investigators examined Head and Neck Squamous Cell Carcinoma (HNSCC) cells and treated the cells with Cox-2 inhibitors (Celecoxib, NS-398 and SC-791) and examined EMT-associated gene products by quantitative real-time PCR and Western blot. The findings demonstrated that the inhibitors upregulated E-cadherin and inhibited its transcriptional repressors such as Snail1. The investigators suggested that the administration of Cox-2 inhibitors may suppress EMT and metastasis via re-expression of E-cadherin.

## Snail1 regulates chemo and immune resistance

Reducing Snail1 expression has proven Snail1’s involvement in tumor resistance to many chemotherapeutic drugs and immunotherapies. In melanoma, Snail1 knockdown, as a result of siRNA treatment, stops both tumor metastasis and immunosuppression. Tumor-specific T cell responses also intensify as a result of this knockdown [144]. Similarly, shRNA treatment induces apoptosis in adriamycin-resistant melanoma cells, and Snail1 reduction leads to cisplatin sensitization in lung adenocarcinoma, head and neck squamous, and ovarian cancers [13],[163]–[165]. Additionally, Snail1 has been implicated in resistance to radiation and paclitaxel in ovarian cancer cell lines as well as protection against 5-fluorouracil and gemcitabine in Panc-1 cells [166],[167].

Snail1 also factors into resistance because of its involvement in survival pathways. Snail1’s activation of MAPK and PI3K survival pathways leads to resistance to serum depletion and TNF-α [168]. The repression of NF-κB and therefore Snail1, its downstream target, sensitizes tumor cells to cisplatin and TNF-related apoptosis-inducing ligand (TRAIL)-induced apoptosis. Treatments with nitric oxide, the proteasome inhibitor NPI-0052, and rituximab all achieve this repression and consequential resistance reversal. These treatments have proven effective in prostate cancers and B-Non-Hodgkin’s Lymphoma, respectively [168]–[171].

Akalay et al. reported that the overexpression of Snail1 in breast cancer cell lines resulted in resistance to CTL-mediated killing and was associated with the EMT phenotype. The resistant cells exhibited amodulation of the formation of the immunologic synapse with CTLs along with the induction of autophagy in the target cells. The findings also showed that the inhibition of autophagy by targeting *Beclin-1* sensitized the EMT cells to CTL killing. Hence, tumor cells’ resistance to CTL is mediated by EMT-induced activation of autophagy-dependent mechanisms [172],[173].

## Chemical inhibitors targeting Snail1

**Figure 4:**
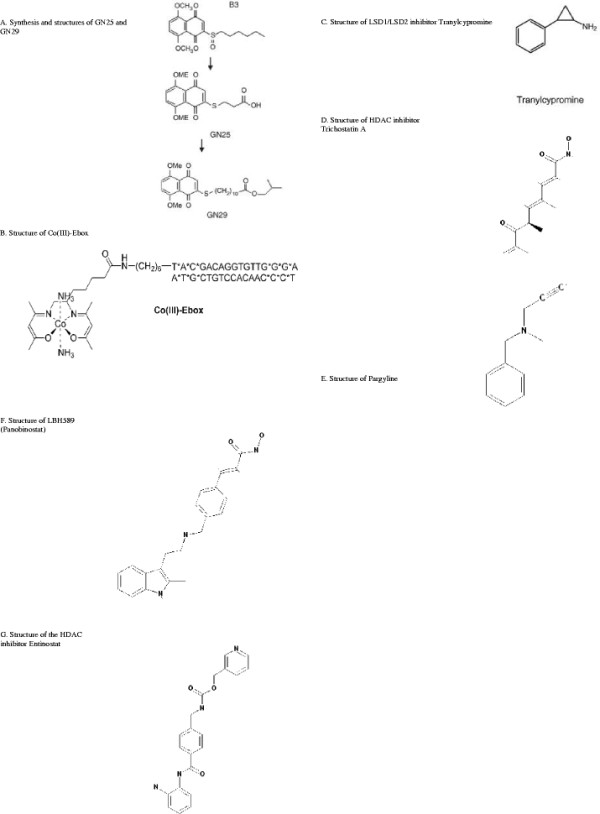
**Structures of chemical inhibitors targeting Snail1. A)** GN 25 and GN 29 [175]**B)** Co(III)-Ebox [176]**C)** Tranylcypromine [183]**D)** Trichostatin A [184]**E)** Pargyline [185]**F)** LBH589 [186] and **G)** Entinostat [187].

Few chemical inhibitors target Snail1 directly. However, Snail1-induced EMT has been successfully abrogated by a select few chemical inhibitors. LSD and HDAC inhibitors, as well as drugs targeting Snail1/p53 and Snail1/E-cadherin interactions, have shown efficacy (Figure 4, Table 4). Their interactions are detailed below.

**Table 4:** Chemical inhibitors that target Snail1-induced EMT

**Name**	**Inhibits**	**Effect**	**Known limitations**	**Reference**
GN25, GN29	Snail/p53 interaction	Reduced proliferation, tumor progression; increased tumor regression	Only effective in K-Ras activated cancer cells and on wild-type p53	[174],[175]
Co(III)-Ebox	Snail/E-cadherin interaction	Increased E-cadherin expression		[176]
Tranylcypromine	LSD1/LSD2	Decreased Snail’s effects on EMT markers		[177]
Trichostatin A	HDAC1/HDAC2	Reversed EMT marker expression		[177]
Pargyline	LSD1	Abrogated Snail-induced EMT		[177]
LBH589	HDAC	Abrogated Snail-induced EMT		[177]
Entinostat	HDAC	Increased E-cadherin and cytokeratin 18 expression, Decreased Twist, Snail, vimentin, N-cadherin; encouraged epithelial morphology; decreased cell migration		[178]

K-Ras-induced Snail1 represses p53, a tumor suppressor encoded by the *TP53* gene, by binding directly and inducing exocytosis [174]. Lee et al. have developed two chemical inhibitors, GN25 and GN29, which prevent this binding and thereby protect p53 and its downstream targets, like p21, from Snail1 [175]. In K-Ras-mutated A549, HCT116, and MKN45 cell lines, both inhibitors were shown to be effective, though GN25 was more so. GN25 and GN29 also inhibited proliferation with more success than did Nutlin-3, which interferes with p53/MDM2 binding. *In vivo* studies indicated that the presence of GN25 reduced tumor progression as well as increased tumor regression. While this mechanism did not have cytotoxic effects on normal cells in this study, it does have some limitations. GN25 only activated wild-type p53 and was not effective in normal fibroblasts and Panc-1 cells. Additionally, this mechanism is effective exclusively in K-Ras-activated cancer cells, not N-Ras/Myc-transformed cells [175].

Harney et al. reported that Co(III)-Ebox, a Co(III) Schiff base complex, interferes with Snail1/E-cadherin binding and thereby inhibits Snail’s repression of the E-cadherin promoter in breast cancer cells [176]. Both the zinc finger region and ability to bind to E-box sequences are critical to this mechanism. With the introduction of Co(III)-Ebox, an increase in E-cadherin gene activity was observed. A 15 nM dose of Co(III)-Ebox achieved maximum results. While Co(III)-Ebox decreased DNA binding, it did not have an effect on Snail1 protein levels in this study [176].

Javaid et al. showed that LSD1, LSD2, and HDAC inhibitors are also effective in countering Snail1-induced EMT [177]. In breast epithelial cells, the LSD1/LSD2 inhibitor Tranylcypromine (TCP) and the HDAC class I and II inhibitor Trichostatin A (TSA) individually decreased Snail1’s effects on epithelial and mesenchymal markers. TSA almost completely reversed EMT markers’ expressions, indicating that HDAC inhibitors can obstruct EMT maintenance in addition to induction. Treatment with both TCP and TSA simultaneously inhibited Snail1-induced EMT, as well as TGF-β-induced EMT. The LSD1 inhibitor Pargyline and the HDAC1, HDAC2, HDAC3, and HDAC6 inhibitor LBH589 were also successful in inhibiting Snail1-induced EMT [177].

Furthermore, Shah et al. found that the HDAC inhibitor entinostat (ENT) reverses Snail1-induced EMT in breast cancer cells [178]. Treating MDA-MB-231 and Hs578T cells with ENT caused an increase in E-cadherin transcription with a concomitant reduction of N-cadherin mRNA. ChIP showed increased E-cadherin promoter activity as well as a reduction in the association of Twist and Snail1. ENT reduced the percentage of CD44^high^/CD24^low^ cells in time and dose dependent manners, and Western blot showed downregulation of Twist and Snail1. Consequently, N-cadherin was reduced, cytokeratin 18 was upregulated, and vimentin was downregulated. Phosphorylation of vimentin increased, and remodeling resulted in a more rounded cell shape. As such, cell morphology became increasingly epithelial and cell migration decreased. ENT thus reverses EMT in triple-negative breast cancer cells, limiting invasive and metastatic potential [178].

Many chemical inhibitors have been developed to target gene products upstream of Snail1. MEK is an attractive target for selective inhibition because of its allosteric binding site, which allows for noncompetitive inhibition, and because all tumors dependent on MAPK signaling are potentially vulnerable to MEK inhibitors [179]. For example, trametinib, a MEK inhibitor, showed higher progression-free and overall survival at six months in phase III trials and was approved by the FDA in May 2013. Selumetinib, which is in phase II trials, has also shown increased PFS and OS [180]. Since PI3K and mTOR have similar catalytic sites, ATP-competitive compounds that target both have been developed in an attempt to increase efficacy. Pre-clinical studies show that dual PI3K/mTOR inhibitors reduce proliferation and induce apoptosis [181].

## Ongoing clinical trials targeting Snail1

Very few ongoing clinical trials relate to Snail1’s role in cancer [182]. In one study, “Polyethylene Glycol 3350 in preventing cancer in patients at risk of colorectal cancer” (NCT00828984), Snail1’s presence will be quantified by immunohistochemistry and RT-PCR. However, Snail1’s role is secondary to EGFR, the true target. The phase II study, which is being conducted by the National Cancer Institute, is listed as recruiting and was last verified in October 2013 [182].

The use of Snail1 as a search term generates a list including collections of tissue samples to study resistance (NCT00880503, NCT00026663) as well as stem cell transplants (NCT01239368, NCT00923845, NCT00074490), but none of these mentions Snail1 specifically in their research descriptions [182]. A phase I HDAC inhibitor study, “A phase I study of belinostat in combination with cisplatin and etoposide in adults with small cell lung carcinoma and other advanced cancers” (NCT00926640), also appears in this list, though it does not cite Snail1 as a target either. The NCI is conducting this study, which was listed as recruiting in its most recent update on March 14, 2014 [182].

## Conclusions and future directions

Snail1, the founding member of the Snail superfamily, is a zinc-finger transcriptional repressor critical to many biological processes. The repression of epithelial markers like E-cadherin, claudins, and mucin-1, in addition to the upregulation of vimentin, fibronectin, and MMPs, facilitates the loss of cell adhesion. Thus, Snail1 confers migratory and invasive properties on epithelial cells. This progression of changing from epithelial cells to a mesenchymal phenotype, known as EMT, is crucial to processes such as gastrulation. Snail1 has also been implicated in cell differentiation and survival.

Snail1 is widely expressed in various cancers, and overexpression is frequently associated with migration, invasion and metastasis. Also correlated with recurrence and a lack of differentiation, Snail1 serves as a poor prognostic indicator in hepatocellular carcinomas, gastric carcinomas, and bladder carcinomas, among others. Therefore, combatting Snail1’s presence could prove pivotal in improving cancer prognoses.

To that end, the development of chemical inhibitors for both Snail1 and targets further upstream has begun [183]–[187]. PI3K, MEK, and mTOR inhibitors are making great strides, and combinations of these prove even more effective. However, many more Snail1-targeting therapies are possible. There are few Snail1-specific chemical inhibitors, and even fewer in clinical trials. Snail1 is ineffective when its nuclear localization is compromised. As such, more can be done to facilitate the phosphorylation and consequential degradation of Snail1 by GSK-3β and proteasomes, respectively. MicroRNA and epigenetic modifications are continually expanding areas of research.

Snail1’s roles in metastasis, recurrence, and resistance make it a novel and pleiotropic target in cancer, and improving our understanding of Snail1 could thus provide new ways of approaching the treatment of metastatic cancer.

## Abbreviations

Akt: Protein kinase B

ALDH: Aldehyde dehydrogenase

β-Trcp: Beta-transducin repeat-containing protein

CDDP: Cisplatin

Co-REST: REST corepressor 1

CSN2: COP9 signalosome 2

CtBP: C-terminal binding protein

EGF: Epidermal growth factor

Egr-1: Early growth response factor-1

ER: Estrogen receptor

ERK: Extracellular signal-regulated kinase

FGF: Fibroblast growth factor

FIGO: International Federation of Gynecology and Obstetrics

GSK-3β: Glycogen synthase kinase-3 beta

HB-EGF: Heparin-binding EGF-like growth factor

HDAC: Histone deacetylase

HGF: Hepatocyte growth factor

HIF-1α: Hypoxia-inducible factor 1-alpha

HMGA2: High-mobility group A2

HRE2: Hypoxia response element-2

IKKα: IκB kinase alpha

ILK: Integrin-linked kinase

IL-6: interleukin 6

JAK: Janus kinase

LEF-1: Lymphoid enhancer-binding factor 1

LOXL2: Lysyl oxidase-like 2

LSD1: Lysine-specific demethylase 1

MAPK: Mitogen-activated protein kinase

MDM2: Mouse double minute 2 homolog

MEK: MAPK kinase

MTA3: Metastasis-associated protein 3

MUPP-1: Multi-PDZ domain protein 1

NF-κB: Nuclear factor kappa-B

NuRD: Nucleosome remodeling deacetylase

Oct-4: Octamer-binding transcription factor 4

PAK1: p21-activated kinase 1

PARP-1: Poly(ADP-ribose) polymerase-1

PI3K: Phosphatidylinositol 3-kinase

PRMT5: Protein arginine methyltransferase 5

RANKL: Receptor activator of nuclear factor kappa-B ligand

STAT3: Signal transducer and activator of transcription 3

Sox-2: Sex determining region Y-box 2

TGF-β1: Transforming growth factor beta 1

TNFα: Tumor necrosis factor alpha

TRAIL: TNF-related apoptosis-inducing ligand

YY1: Yin Yang 1

ZEB1/2: Zinc finger E-box-binding homeobox 1/2

ZO-1/2: Zonula occludens protein 1/2

## Competing interests

The authors declare that they have no competing interests.

## Authors’ contributions

SK was responsible for reviewing the literature, summarizing data and preparing a draft of the manuscript. BB conceptualized and developed an outline for the manuscript as well as edited the manuscript for publication. Both authors read and approved the final manuscript.

## References

[B1] Nieto MA The snail superfamily of zinc-finger transcription factors Nat Rev Mol Cell Biol 2002 3 155 166 11994736 10.1038/nrm757

[B2] Boulay J Dennefeld C Alberga A The *Drosophila* developmental gene snail encodes a protein with nucleic acid binding fingers Nature 1987 330 395 398 3683556 10.1038/330395a0

[B3] Manzanares M Locascio A Nieto MA The increasing complexity of the snail gene superfamily in metazoan evolution Trends Genet 2001 17 178 181 11275308 10.1016/s0168-9525(01)02232-6

[B4] Grau Y Carteret C Simpson P Mutations and chromosomal rearrangements affecting the expression of snail, a gene involved in embryonic patterning in *Drosophila melanogaster* Genetics 1984 108 347 360 17246230 10.1093/genetics/108.2.347 PMC1202410

[B5] Nusslein-Volhard C Weischaus E Kluding H Mutations affecting the pattern of the larval cuticle in Drosophila melanogaster. I. Zygotic loci on the second chromosome Wilheim Roux’s Arch Dev Biol 1984 193 267 282 10.1007/BF00848156 28305337

[B6] Twigg S Wilkie AOM Characterization of the human snail (SNAI1) gene and exclusion as a major disease gene in craniosynostosis Hum Genet 1999 105 320 326 10543399 10.1007/s004399900143

[B7] Paznekas W Okajima K Schertzer M Wood S Jabs E Genomic organization, expression, and chromosome location of the human snail gene (SNAI1) and a related processed pseudogene (SNAI1P) Genomics 1999 62 42 49 10585766 10.1006/geno.1999.6010

[B8] Barrallo-Gimeno A Nieto MA Evolutionary history of the snail/scratch superfamily Trends Genet 2009 25 248 252 19427053 10.1016/j.tig.2009.04.001

[B9] http://www.uniprot.org/uniprot/O95863 Human Snail1: sequence retrieved from and alignments run through NIH BLAST http://blast.st-va.ncbi.nlm.nih.gov/Blast.cgi.

[B10] Kalluri R Weinberg R The basics of epithelial-mesenchymal transition J Clin Invest 2009 119 1420 1428 19487818 10.1172/JCI39104 PMC2689101

[B11] Carver EA Jiang R Gridley T The mouse snail gene encodes a key regulator of the epithelial-mesenchymal transition Mol Cell Biol 2001 21 8184 8188 11689706 10.1128/MCB.21.23.8184-8188.2001 PMC99982

[B12] Barrallo-Gimeno A Nieto MA The Snail genes as inducers of cell movement and survival: implications in development and cancer Development 2005 132 3151 3161 15983400 10.1242/dev.01907

[B13] Kajita M McClinic K Wade P Aberrant expression of the transcription factors Snail and Slug alters the response to genotoxic stress Mol Cell Biol 2004 24 7559 7566 15314165 10.1128/MCB.24.17.7559-7566.2004 PMC506998

[B14] Mani S Guo W Liao MJ Eaton E Ayyanan A Zhou AY Brooks M Reinhard F Zhang CC Shipitsin M Campbell LL Polyak K Brisken C Yang J Weinberg RA The epithelial-mesenchymal transition generates cells with properties of stem cells Cell 2008 133 704 715 18485877 10.1016/j.cell.2008.03.027 PMC2728032

[B15] Zhou W Lv R Qi W Wu D Xu Y Liu W Mou Y Wang L Snail contributes to the maintenance of stem cell-like phenotype cells in human pancreatic cancer PLoS One 2014 9 e87409 24489910 10.1371/journal.pone.0087409 PMC3906155

[B16] Wang H Zhang G Zhang H Zhang F Zhou BP Ning F Wang HS Cai SH Du J Acquisition of epithelial-mesenchymal transition phenotype and cancer stem cell-like properties in cisplatin-resistant lung cancer cells through AKT/β-catenin/Snail signaling pathway Eur J Pharmacol 2014 723 156 166 24333218 10.1016/j.ejphar.2013.12.004

[B17] Majmundar AJ Wong WJ Simon MC Hypoxia-inducible factors and the response to hypoxic stress Mol Cell 2010 40 294 309 20965423 10.1016/j.molcel.2010.09.022 PMC3143508

[B18] Peinado H Del Carmen Iglesias-de la Cruz M Olmeda D Csiszar K Fong KS Vega S Nieto MA Cano A Portillo F A molecular role for lysyl oxidase-like 2 enzyme in snail regulation and tumor progression EMBO J 2005 24 3446 3458 16096638 10.1038/sj.emboj.7600781 PMC1276164

[B19] Zhu GH Huang C Feng ZZ Lv XH Qiu ZJ Hypoxia-induced snail expression through transcriptional regulation by HIF-1alpha in pancreatic cancer cells Dig Dis Sci 2013 58 3503 3515 23979441 10.1007/s10620-013-2841-4

[B20] Barbera MJ Puig I Dominguez D Julien-Grille S Guaita-Esteruelas S Peiro S Baulida J Franci C Dedhar S Larue L Garcia de Herreros A Regulation of snail transcription during epithelial to mesenchymal transition of tumor cells Oncogene 2004 23 7345 7354 15286702 10.1038/sj.onc.1207990

[B21] Brandl M Seidler B Haller F Adamski J Schmid RM Saur D Schneider G IKKalpha controls canonical TGFBeta-SMAD signaling to regulate genes expressing snail and slug during EMT in Panc1 cells J Cell Sci 2010 123 4231 4239 21081648 10.1242/jcs.071100

[B22] Thuault S Tan EJ Peinado H Cano A Heldin CH Moustakas A HMGA2 and Smads co-regulate SNAIL1 expression during induction of epithelial-to-mesenchymal transition J Biol Chem 2008 283 33437 33446 18832382 10.1074/jbc.M802016200 PMC2662269

[B23] McPhee T McDonald P Oloumi A Dedhar S Integrin-linked kinase regulates E-Cadherin expression through PARP-1 Dev Dyn 2008 237 2737 2747 18773488 10.1002/dvdy.21685

[B24] Yadav A Kumar B Datta J Teknos T Kumar P IL-6 promotes head and neck tumor metastasis by inducing epithelial-mesenchymal transition via the JAK-STAT3-SNAIL signaling pathway Mol Cancer Res 2011 9 1658 1667 21976712 10.1158/1541-7786.MCR-11-0271 PMC3243808

[B25] Zhang XH Liang X Wang TS Liang XH Zuo RJ Deng WB Zhang ZR Qin FN Zhao ZA Yang ZM Heparin-binding epidermal growth factor-like growth factor (HB-EGF) induction on Snail expression during mouse decidualization Mol Cell Endocrinol 2013 381 272 279 23994020 10.1016/j.mce.2013.08.011

[B26] Li X Deng W Lobo-Ruppert S Ruppert J Gli1 acts through Snail and E-Cadherin to promote nuclear signaling by Beta-catenin Oncogene 2007 26 4489 4498 17297467 10.1038/sj.onc.1210241 PMC2233601

[B27] Fujita N Jaye D Kajita M Geigerman C Moreno C Wade P MTA3, a Mi-2/NuRD complex subunit, regulates an invasive growth pathway in breast cancer Cell 2003 113 207 219 12705869 10.1016/s0092-8674(03)00234-4

[B28] Dhasarathy A Kajita M Wade P The transcription factor snail mediates epithelial to mesenchymal transitions by repression of estrogen receptor-alpha Mol Endocrinol 2007 21 2907 2918 17761946 10.1210/me.2007-0293 PMC2668600

[B29] Grotegut S von Schweinitz D Christofori G Lehembre F Hepatocyte growth factor induces cell scattering through MAPK/Egr-1-mediated upregulation of Snail EMBO J 2006 25 3534 3545 16858414 10.1038/sj.emboj.7601213 PMC1538570

[B30] Palmer M Majumder P Cooper J Yoon H Wade P Boss J Yin Yang 1 regulates the expression of Snail through a distal enhancer Mol Cancer Res 2009 7 221 229 19208738 10.1158/1541-7786.MCR-08-0229 PMC2819842

[B31] Peiro S Escriva M Puig I Barbera MJ Dave N Herranz N Larriba MJ Takkunen M Franci C Munoz A Virtanen I Baulida J Garcia de herreros A Snail1 transcriptional repressor binds to its own promoter and controls its expression Nucleic Acids Res 2006 34 2077 2084 16617148 10.1093/nar/gkl141 PMC1440880

[B32] Kim NH Kim HS Li XY Lee I Choi HS Kang SE Cha SY Ryu JK Yoon D Fearon ER Rowe RG Lee S Maher CA Weiss SJ Yook JI A p53/miRNA-34 axis regulates Snail1-dependent cancer cell epithelial-mesencymal transition J Cell Biol 2011 195 417 433 22024162 10.1083/jcb.201103097 PMC3206336

[B33] Zhou BP Deng J Xia W Xu J Li Y Gunduz M Hung MC Dual regulation of Snail by GSK-3beta-mediated phosphorylation in control of epithelial-mesenchymal transition Nat Cell Biol 2004 6 931 940 15448698 10.1038/ncb1173

[B34] Katoh M Katoh M Cross-talk of WNT and FGF signaling pathways at GSK3beta to regulate beta-catenin and SNAIL signaling cascades Cancer Biol Ther 2006 5 1059 1064 16940750 10.4161/cbt.5.9.3151

[B35] Vinas-Castells R Beltran M Valls G Gomez I Garcia JM Montserrat-Sentis B Baulida J Bonilla F Garcia de herreros A Diaz VM The hypoxia-controlled FBXL14 ubiquitin ligase targets SNAIL1 for proteasome degradation J Biol Chem 2010 285 3794 3805 19955572 10.1074/jbc.M109.065995 PMC2823521

[B36] Yang Z Rayala S Nguyen D Vadlmudi R Chen S Kumar R Pak1 phosphorylation of snail, a master regulator of epithelial-to-mesenchhyme transition, modulates snail’s subcellular localization and functions Cancer Res 2005 65 3179 3184 15833848 10.1158/0008-5472.CAN-04-3480

[B37] Dominguez D Montserrat-Sentis B Virgos-Soler A Guaita S Grueso J Porta M Puig I Baulida J Franci C Garcia de Herreros A Phosphorylation regulates the subcellular location and activity of the snail transcriptional repressor Mol Cell Biol 2003 23 5078 5089 12832491 10.1128/MCB.23.14.5078-5089.2003 PMC162233

[B38] Ko H Kim H Kim N Lee S Kim K Hong S Yook J Nuclear localization signals of the E-Cadherin transcriptional repressor Snail Cells Tissues Organs 2007 185 66 72 17587810 10.1159/000101305

[B39] Wu Y Deng J Rychahou PG Qiu S Evers BM Zhou BP Stabilization of snail by NFkappaB is required for inflammation-induced cell migration and invasion Cancer Cell 2009 15 416 428 19411070 10.1016/j.ccr.2009.03.016 PMC2881229

[B40] Wu Y Zhou BP Snail: more than EMT Cell Adhes Migrat 2010 4 199 203 10.4161/cam.4.2.10943 PMC2900613 20168078

[B41] Yook JI Li XY Ota I Fearon ER Weiss SJ Wnt-dependent regulation of the E-cadherin repressor snail J Biol Chem 2005 280 11740 11748 15647282 10.1074/jbc.M413878200

[B42] Zhang JP, Zeng C, Xu L, Gong J, Fang JH, Zhuang SM: **MicroRNA-148a suppresses the epithelial-mesenchymal transition and metastasis of hepatoma cells by targeting Met/Snail signaling.** *Oncogene* 2013, Epub ahead of print. 10.1038/onc.2013.369 24013226

[B43] Tsubaki M Komai M Fujimoto SI Itoh T Imano M Sakamoto K Shimaoka H Takeda T Ogawa N Mashimo K Fujiwara D Mukai J Sakaguchi K Satou T Nishida S Activation of NF-κB by the RANKL/RANK system up-regulates snail and twist expressions and induces epithelial-to-mesenchymal transition in mammary tumor cell lines J Exp Clin Cancer Res 2013 32 62 24011086 10.1186/1756-9966-32-62 PMC3847095

[B44] Julien S Puig I Caretti E Bonaventure J Nelles L van Roy F Dargemont C de Herreros AG Bellacosa A Larue L Activation of NF-κB by Akt upregulates Snail expression and induces epithelium mesenchyme transition Oncogene 2007 26 7445 7456 17563753 10.1038/sj.onc.1210546

[B45] Cheng JC Chang HM Leung P TGF-Beta1 inhibits trophoblast cell invasion by inducing snail-mediated down-regulation of ve-cadherin J Biol Chem 2013 288 33181 33192 24106276 10.1074/jbc.M113.488866 PMC3829165

[B46] Horiguchi K Shirakihara T Nakano A Imamura T Miyazono K Saitoh M Role of Ras signaling in the induction of snail by transforming growth factor-beta J Biol Chem 2009 284 245 253 19010789 10.1074/jbc.M804777200

[B47] Wu Y Evers BM Zhou BP Small C-terminal domain phosphatase enhances snail activity through dephosphorylation J Biol Chem 2009 284 640 648 19004823 10.1074/jbc.M806916200 PMC2610500

[B48] Jiang GM Wang HS Zhang F Zhang KS Liu ZC Fang R Wang H Cai SH Du J Histone deacetylase inhibitor induction of epithelial-mesenchymal transitions via up-regulation of Snail facilitates cancer progression Biochim Biophys Acta 1833 2013 663 671 10.1016/j.bbamcr.2012.12.002 23246564

[B49] Takeichi M Functional correlation between cell adhesive properties and some cell surface proteins J Cell Biol 1977 75 464 474 264120 10.1083/jcb.75.2.464 PMC2109947

[B50] Berx G Staes K van Hengel J Molemans F Bussemakers M von Bokhoven A van Roy F Cloning and characterization of the human invasion suppressor gene E-cadherin (CDH1) Genomics 1995 26 281 289 7601454 10.1016/0888-7543(95)80212-5

[B51] Van Roy F Berx G The cell-cell adhesion molecule E-cadherin Cell Mol Life Sci 2008 65 3756 3788 18726070 10.1007/s00018-008-8281-1 PMC11131785

[B52] Takeichi M Matsunami H Inoue T Kimura Y Suzuki S Tanaka T Roles of cadherins in patterning of the developing brain Dev Neurosci 1997 19 86 87 9078437 10.1159/000111189

[B53] Vestweber D Kemler R Identification of a putative cell adhesion domain of uvomorulin EMBO J 1985 4 3393 3398 2419126 10.1002/j.1460-2075.1985.tb04095.x PMC554675

[B54] Cano A Perez-Moreno MA Rodrigo I Locascio A Blanco MJ del Barrio MG Portillo F Nieto MA The transcription factor Snail controls epithelial-mesenchymal transitions by repressing E-cadherin expression Nat Cell Biol 2000 2 76 83 10655586 10.1038/35000025

[B55] Larue L Ohsugi M Hirchenhain J Kemler R E-cadherin null mutant embryos fail to form a trophectoderm epithelium Proc Natl Acad Sci U S A 1994 91 8263 8267 8058792 10.1073/pnas.91.17.8263 PMC44586

[B56] Dong C Wu Y Yao J Wang Y Yu Y Rychahou P Evers B Zhou B G9a interacts with snail and is critical for snail-mediated E-cadherin repression in human breast cancer J Clin Investig 2012 122 1469 1486 22406531 10.1172/JCI57349 PMC3314447

[B57] Hou Z Peng H Ayyanathan K Yan KP Langer EM Longmore GD Rauscher FJ III The LIM protein AJUBA recruits protein arginine methyltransferase 5 to mediate SNAIL-dependent transcriptional repression Mol Cell Biol 2008 28 3198 3207 18347060 10.1128/MCB.01435-07 PMC2423142

[B58] Shi Y Whetstine JR Dynamic regulation of histone lysine methylation by demethylases Mol Cell 2007 25 1 14 17218267 10.1016/j.molcel.2006.12.010

[B59] Peinado H Ballestar E Esteller M Cano A Snail mediates E-cadherin repression by the recruitment of the Sin3A/histone deacetylase 1 (HDAC1)/HDAC2 complex Mol Cell Biol 2004 24 306 319 14673164 10.1128/MCB.24.1.306-319.2004 PMC303344

[B60] Lin Y Wu Y Li J Dong C Ye X Chi YI Evers BM Zhou BP The SNAG domain of Snail1 functions as a molecular hook for recruiting lysine-specific demethylase 1 EMBO J 2010 29 1803 1816 20389281 10.1038/emboj.2010.63 PMC2885925

[B61] Dong C Wu Y Wang Y Wang C Kang T Rychahou PG Chi YI Evers BM Zhou BP Interaction with Suv39H1 is critical for Snail-mediated E-cadherin repression in breast cancer Oncogene 2013 32 1351 1362 22562246 10.1038/onc.2012.169 PMC3703513

[B62] Yeung K Seitz T Li S Janosch P McFerran B Kaiser C Fee F Katsanakis KD Rose DW Mischak H Sedivy JM Kolch W Suppression of Raf-1 kinase activity and MAP kinase signaling by RKIP Nature 1999 401 173 177 10490027 10.1038/43686

[B63] Yeung K Rose DW Dhillon AS Yaros D Gusafsson M Chatterjee D McFerran B Wyche J Kolch W Sedivy JM Raf kinase inhibitor protein interacts with NF-kappaB-inducing kinase and TAK1 and inhibits NF-kappaB activation Mol Cell Biol 2001 21 7201 7217 10.1128/MCB.21.21.7207-7217.2001 PMC99896 11585904

[B64] Chatterjee D Bai Y Wang Z Beach S Mott S Roy R Braastad C Sun Y Mukhopadhyay A Aggarwal BB Darnowski J Pantazis P Wyche J Fu Z Kitagwa Y Keller ET Sedivy JM Yeung KC RKIP sensitizes prostate and breast cancer cells to drug-induced apoptosis J Biol Chem 2004 279 17515 17523 14766752 10.1074/jbc.M313816200

[B65] Park S Yeung ML Beach S Shields JM Yeung KC RKIP downregulates B-Raf kinase activity in melanoma cancer cells Oncogene 2005 24 3535 3540 15782137 10.1038/sj.onc.1208435

[B66] Al-Mulla F Hagan S Behbehani AI Bitar MS George SS Going JJ Garcia JJ Scott L Fyfe N Murray GI Kolch W Raf kinase inhibitor protein expression in a survival analysis of colorectal cancer patients J Clin Oncol 2006 24 5672 5679 17179102 10.1200/JCO.2006.07.5499

[B67] Fu Z Kitagawa Y Shen R Shah R Mehra R Rhodes D Keller PJ Mizokami A Dunn R Chinnaiyan AM Yao Z Keller ET Metastasis suppressor gene Raf kinase inhibitor protein (RKIP) is a novel prognostic marker in prostate cancer Prostate 2005 66 248 256 16175585 10.1002/pros.20319

[B68] Beach S Tang H Park S Dhillon AS Keller ET Kolch W Yeung KC Snail is a repressor of RKIP transcription in metastatic prostate cancer cells Oncogene 2008 27 2243 2248 17952120 10.1038/sj.onc.1210860 PMC2933472

[B69] Vazquez F Devreotes P Regulation of PTEN Function as a PIP3 Gatekeeper through Membrane Cell Cycle 2006 5 1523 1527 16861931 10.4161/cc.5.14.3005

[B70] Escriva M Peiro S Herranz H Villagrasa P Dave N Montserrat-Sentis B Murray SA Franci C Gridley T Virtanen I Garcia de herreros A Repression of PTEN Phosphatase by Snail1 Transcriptional Factor during Gamma Radiation-Induced Apoptosis Mol Cell Biol 2008 28 1528 1540 18172008 10.1128/MCB.02061-07 PMC2258777

[B71] Stambolic V MacPherson D Sas D Lin Y Snow B Jang Y Benchimol S Mak TW Regulation of PTEN transcription by p53 Mol Cell 2001 8 317 325 11545734 10.1016/s1097-2765(01)00323-9

[B72] Yamada KM Araki M Tumor suppressor PTEN: modulator of cell signalling, growth, migration and apoptosis J Cell Sci 2002 114 2375 2382 11559746 10.1242/jcs.114.13.2375

[B73] Furuse M Hirase T Itoh M Nagafuchi A Yonemura S Tsukita S Tsukita S Occludin: a novel integral membrane protein localizing at tight junctions J Cell Biol 1993 123 1777 1788 8276896 10.1083/jcb.123.6.1777 PMC2290891

[B74] Ando-Akatsuka Y Saitou M Hirase T Kishi M Sakakibara A Itoh M Yonemura S Furuse M Tsukita S Interspecies diversity of the occludin sequence: cDNA cloning of human, mouse, dog, and rat-kangaroo homologues J Cell Biol 1996 133 43 47 8601611 10.1083/jcb.133.1.43 PMC2120780

[B75] Ikenouchi J Matsuda M Furuse M Tsukita S Regulation of tight junctions during the epithelium-mesenchyme transition: direct repression of the gene expression of claudins/occludin by Snail J Cell Sci 2003 116 1959 1967 12668723 10.1242/jcs.00389

[B76] Findley M Koval M Regulation and roles for claudin-family tight junction proteins IUBMB Life 2009 61 431 437 19319969 10.1002/iub.175 PMC2708117

[B77] Martinez-Estrada O Culleres A Vilaro S The transcription factors Slug and Snail act as repressors of Claudin-1 expression in epithelial cells Biochem J 2006 394 449 457 16232121 10.1042/BJ20050591 PMC1408675

[B78] Martin T Jiang W Loss of tight junction barrier function and its role in cancer metastasis BBA Biomembranes 2009 1788 872 891 19059202 10.1016/j.bbamem.2008.11.005

[B79] Zaretsky J Barnea I Aylon Y Gorivodsky M Wreschner D Keydar I MUC1 gene overexpressed in breast cancer: structure and transcriptional activity of the MUC1 promoter and role of estrogen receptor alpha (ERalpha) in regulation of the MUC1 gene expression Mol Cancer 2006 5 57 17083744 10.1186/1476-4598-5-57 PMC1636664

[B80] Brayman M Thathiah A Carson D MUC1: a multifunctional cell surface component of reproductive tissue epithelia Reprod Biol Endocrinol 2004 2 4 14711375 10.1186/1477-7827-2-4 PMC320498

[B81] Hollingsworth M Swanson B Mucins in cancer: protection and control of the cell surface Nat Rev Cancer 2004 4 45 60 14681689 10.1038/nrc1251

[B82] Gendler S Spicer A Epithelial mucin genes Annu Rev Physiol 1995 57 607 634 7778880 10.1146/annurev.ph.57.030195.003135

[B83] Guaita S Puig I Franci C Garrido M Dominguez D Batlle E Sancho E Dedhar S De Herreros AG Baulida J Snail induction of epithelial to mesenchymal transition in tumor cells is accompanied by MUC1 repression and ZEB1 expression J Biol Chem 2002 277 39209 39216 12161443 10.1074/jbc.M206400200

[B84] Sanchez-Tillo E Lazaro A Torrent R Cuatrecasas M Vaquero EC Castells A Engel P Postigo A ZEB1 represses E-cadherin and induces an EMT by recruiting the SWI/SNF chromatin-remodeling protein BRG1 Oncogene 2010 29 3490 3500 20418909 10.1038/onc.2010.102

[B85] Satelli A Li S Vimentin in cancer and its potential as a molecular target for cancer therapy Cell Mol Life Sci 2011 68 3033 3046 21637948 10.1007/s00018-011-0735-1 PMC3162105

[B86] Lilienbaum A Paulin D Activation of the human vimentin gene by the Tax human T-cell leukemia virus. I. Mechanisms of regulation by the NF-kappa B transcription factor J Biol Chem 1993 268 2180 2188 8420985

[B87] Wu Y Zhang X Salmon M Lin X Zehner ZE TGFbeta1 regulation of vimentin gene expression during differentiation of the C2C12 skeletal myogenic cell line requires Smads, AP-1 and Sp1 family members Biochim Biophys Acta 2007 1773 427 439 17270292 10.1016/j.bbamcr.2006.11.017 PMC1855268

[B88] Zhu QS Rosenblatt K Huang KL Lahat G Brobey R Bolshakov S Nguyen T Ding Z Belousov R Bill K Luo X Lazar A Dicker A Mills GB Hung MC Lev D Vimentin is a novel AKT1 target mediating motility and invasion Oncogene 2011 30 457 470 20856200 10.1038/onc.2010.421 PMC3010301

[B89] Gilles C Polette M Mestdagt M Nawrocki-Raby B Ruggeri P Birembaut P Foidart JM Transactivation of vimentin by beta-catenin in human breast cancer cells Cancer Res 2003 63 2658 2664 12750294

[B90] Lang SH Hyde C Reid IN Hitchcock IS Hart CA Bryden AA Villette JM Stower MJ Maitland NJ Enhanced expression of vimentin in motile prostate cell lines and in poorly differentiated and metastatic prostate carcinoma Prostate 2002 52 253 263 12210485 10.1002/pros.10088

[B91] Zhao Y Yan Q Long X Chen X Wang Y Vimentin affects the mobility and invasiveness of prostate cancer cells Cell Biochem Funct 2008 26 571 577 18464297 10.1002/cbf.1478

[B92] Hynes RO Yamada KM Fibronectins: multifunctional modular glycoproteins J Cell Biol 1982 95 369 377 6128348 10.1083/jcb.95.2.369 PMC2112946

[B93] Mosher DF Fibronectin 1989 Academic Press, Inc., San Diego

[B94] Pankov R Yamada K Fibronectin at a glance J Cell Sci 2002 115 3861 3863 12244123 10.1242/jcs.00059

[B95] Benecky MJ Kolvenback CG Amrani DL Mosesson MN Evidence that binding to the carboxyl-terminal heparin-binding domain (HepII) dominates the interaction between plasma fibronectin and heparin Biochem 1988 27 7565 7571 3207688 10.1021/bi00419a058

[B96] Ingham KC Brew SA Atha DH Interaction of heparin with fibronectin and isolated fibronectin domains Biochem J 1990 272 605 611 2268289 10.1042/bj2720605 PMC1149751

[B97] Mostafavi-Pour Z Askari JA Whittard JD Humphries MJ Identification of a novel heparin-binding site in the alternatively spliced IIICS region of fibronectin: roles of integrins and proteoglycans in cell adhesion to fibronectin splice variants Matrix Biol 2001 20 63 73 11246004 10.1016/s0945-053x(00)00131-1

[B98] Liao YF Gotwals PJ Koteliansky VE Sheppard D Van De Water L The EIIIA segment of fibronectin is a ligand for integrins α9β1 andα 4β1 providing a novel mechanism for regulating cell adhesion by alternative splicing J Biol Chem 2002 277 14467 14474 11839764 10.1074/jbc.M201100200

[B99] Erat MC Sladek B Campbell ID Vakonakis I Structural analysis of collagen type I interactions with human fibronectin reveals a cooperative binding mode J Biol Chem 2013 288 17441 17450 23653354 10.1074/jbc.M113.469841 PMC3682544

[B100] George EL Georges-Labouesse EN Patel-King RS Rayburn H Hynes RO Defects in mesoderm, neural tube and vascular development in mouse embryos lacking fibronectin Development 1993 119 1079 1091 8306876 10.1242/dev.119.4.1079

[B101] Moll R Franke WW Schiller DL Geiger B Krepler R The catalog of human cytokeratins: patterns of expression in normal epithelia, tumors and cultured cells Cell 1982 31 11 24 6186379 10.1016/0092-8674(82)90400-7

[B102] Fuchs E Cleveland DW A structural scaffolding of intermediate filaments in health and disease Science 1998 279 514 519 9438837 10.1126/science.279.5350.514

[B103] Coulombe PA Omary MB ‘Hard’ and ‘soft’ principles defining the structure, function and regulation of keratin intermediate filaments Curr Opin Cell Biol 2002 14 110 122 11792552 10.1016/s0955-0674(01)00301-5

[B104] Galarneau L Loranger A Gilbert S Marceau N Keratins modulate hepatic cell adhesion, size and G1/S transition Exp Cell Res 2007 313 179 194 17112511 10.1016/j.yexcr.2006.10.007

[B105] Oshima RG Baribault H Caulín C Oncogenic regulation and function of keratins 8 and 18 Cancer Metastasis Rev 1996 15 445 471 9034603 10.1007/BF00054012

[B106] Lin MH Liu SY Su HJ Liu YC Functional role of matrix metalloproteinase 28 in the oral squamous cell carcinoma Oral Oncol 2006 42 907 913 16730219 10.1016/j.oraloncology.2005.12.012

[B107] Birkedal-Hansen H Moore WG Bodden MK Windsor LJ Birkedal-Hansen B DeCarlo A Engler JA Matrix Metalloproteinases: a review Crit Rev Oral Biol Med 1993 4 197 250 8435466 10.1177/10454411930040020401

[B108] Senior RM Griffin GL Fliszar CJ Shapiro SD Goldberg GI Welgus HG Human 92- and 72- kilodalton type IV collagenases are elastases J Biol Chem 1991 266 7870 7875 1850424

[B109] Seltzer JL Adams SA Grant GA Eisen AZ Purification and properties of a gelatin-specific neutral protease from human skin J Biol Chem 1981 256 4662 4668 6260809

[B110] Seltzer JL Eisen AZ Bauer EA Morris NP Glanville RW Burgeson RE Cleavage of type VII collagen by interstitial collagenase and type IV collagenase (Gelatinase) derived from human skin J Biol Chem 1989 264 3822 3826 2537292

[B111] Gadher SJ Schmid TM Heck LW Woolley DE Cleavage of collagen type X by human synovial collagenase and neutrophil elastase Matrix 1989 9 109 115 2542740 10.1016/s0934-8832(89)80028-9

[B112] Huhtala P Tuuttila A Chow LT Lohi J Keski-Oja J Tryggvason K Complete structure of the human gene for 92-kDa type IV collagenase. Divergent regulation of expression for the 92- and 72-kilodalton enzyme genes in HT-1080 cells J Biol Chem 1991 266 16485 16490 1653238

[B113] Qiao B Johnson N Gao J Epithelial-mesenchymal transition in oral squamous cell carcinoma triggered by transforming growth factor-β1 is Snail family-dependent and correlates with matrix metalloproteinase-2 and -9 expressions Int J Oncol 2010 37 663 668 20664935 10.3892/ijo_00000715

[B114] Liotta LA Tryggvason K Garbisa S Hart I Foltz CM Shafie S Metastatic potential correlates with enzymic degradation of basement membrane collagen Nature 1980 284 67 68 6243750 10.1038/284067a0

[B115] Garbisa S Pozzati R Muschel RJ Saffiotti U Ballin M Goldfarb RH Khoury G Liotta LA Secretion of type IV collagenolytic protease and metastatic phenotype: induction by transfection with C-Ha-ras but not C-Ha-ras plus Ad2-Ela Cancer Res 1987 47 1523 1528 3028610

[B116] Nakajima M Welch DR Belloni PN Nicholson GL Degradation of basement membrane type IV collagen and lung subendothelial matrix by rat mammary adenocarcinoma cell clones of differing metastatic potentials Cancer Res 1987 47 4869 4876 3621180

[B117] Bernhard EJ Muschel RJ Hughes EN Mr 92,000 gelatinase release correlates with the metastatic phenotype in transformed rat embryo cells Cancer Res 1990 50 3872 3877 2162246

[B118] Mahabir R Tanino M Elmansuri A Wang L Kimura T Itoh T Ohba Y Nishihara H Shirato H Tsuda M Tanaka S Sustained elevation of Snail promotes glial-mesenchymal transition after irradiation in malignant glioma Neuro Oncol 2013 0 1 15 10.1093/neuonc/not239 PMC3984547 24357458

[B119] Porfiri E Rubinfeld B Albert I Hovanes K Waterman M Polakis P Induction of a β-catenin-LEF-1 complex by wnt-1 and transforming mutants of β-catenin Oncogene 1997 15 2833 2839 9419974 10.1038/sj.onc.1201462

[B120] Rubinfeld B Robbins P El-Gamil M Albert I Porfiri E Polakis P Stabilization of β-catenin by genetic defects in melanoma cell lines Science 1997 275 1790 1792 9065403 10.1126/science.275.5307.1790

[B121] Jamora C DasGupta R Kocieniewski P Fuchs E Links between signal transduction, transcription and adhesion in epithelial bud development Nature 2003 422 317 322 12646922 10.1038/nature01458 PMC2424170

[B122] Kim K Lu Z Hay ED Direct evidence for a role of betacatenin/LEF-1 signalling pathway in the induction of EMT Cell Biol Int 2002 26 463 476 12095232 10.1006/cbir.2002.0901

[B123] Waterman ML Lymphoid enhancer factor/T cell factor expression in colorectal cancer Cancer Metastasis Rev 2004 23 41 52 15000148 10.1023/a:1025858928620

[B124] Medici D Hay E Goodenough D Cooperation between Snail and LEF-1 transcription factors is essential for TGF-β1-induced epithelial-mesenchymal transition Mol Biol Cell 2006 17 1871 1879 16467384 10.1091/mbc.E05-08-0767 PMC1415320

[B125] De Craene B van Roy F Berx G Unraveling signaling cascades for the Snail family of transcription factors Cell Signal 2005 17 535 547 15683729 10.1016/j.cellsig.2004.10.011

[B126] Elston CW Ellis IO Pathological prognostic factors in breast cancer. I. The value of histological grade in breast cancer: experience with long-term follow-up Histopathology 1991 19 403 410 1757079 10.1111/j.1365-2559.1991.tb00229.x

[B127] Dieterich M Goodman SN Rojas-Corona RR Emralino AB Jimenez-Joseph D Sherman ME Multivariate analysis of prognostic features in malignant pleural effusions from breast cancer patients Acta Cytol 1994 38 945 952 7992584

[B128] Blanco MJ Moreno-Bueno G Sarrio D Locascio A Cano A Palacios J Nieto MA Correlation of Snail expression with histological grade and lymph node status in breast carcinomas Oncogene 2002 21 3241 3246 12082640 10.1038/sj.onc.1205416

[B129] Elloul S Bukholt Elstrand M Nesland JM Trope CG Kvalheim G Goldberg I Reich R Davidson B Snail, Slug, and Smad-interacting protein 1 as novel parameters of disease aggressiveness in metastatic ovarian and breast carcinoma Cancer 2005 103 1631 1643 15742334 10.1002/cncr.20946

[B130] Jiao W Miyazaki K Kitajima Y Inverse correlation between E-cadherin and Snail expression in hepatocellular carcinoma cell lines in vitro and in vivo Br J Cancer 2002 86 98 101 11857019 10.1038/sj.bjc.6600017 PMC2746537

[B131] Miyoshi A Kitajima Y Miyazaki K Snail accelerates cancer invasion by upregulating MMP expression and is associated with poor prognosis of hepatocellular carcinoma Br J Cancer 2005 92 252 258 15668718 10.1038/sj.bjc.6602266 PMC2361838

[B132] Woo HY Min AL Choi JY Bae SH Yoon SK Jung CK Clinicopathologic significance of the expression of Snail in hepatocellular carcinoma Korean J Hepatol 2011 17 12 18 21494073 10.3350/kjhep.2011.17.1.12 PMC3304621

[B133] Elloul S Silins I Trope CG Benshushan A Davidson B Reich R Expression of E-cadherin transcriptional regulators in ovarian carcinoma Virchows Arch 2006 449 520 528 17024425 10.1007/s00428-006-0274-6

[B134] Rosiavitz E Becker I Specht K Fricke E Luber B Busch R Hofler H Becker KF Differential expression of the epithelial-mesenchymal transition regulators Snail, SIP1, and Twist in gastric cancer Am J Pathol 2002 161 1881 1891 12414534 10.1016/S0002-9440(10)64464-1 PMC1850763

[B135] Shin NR Jeong EH Choi CI Moon HJ Kwon CH Chu IS Kim GH Jeon TY Kim DH Lee JH Park do Y Overexpression of Snail is associated with lymph node metastasis and poor prognosis in patients with gastric cancer BMC Cancer 2012 12 521 23151184 10.1186/1471-2407-12-521 PMC3552976

[B136] Yokoyama K Kamata N Hayashi E Hoteiya T Ueda N Fujimoto R Nagayama M Reverse correlation of E-cadherin and snail expression in oral squamous cell carcinoma cells in vitro Oral Oncol 2001 37 65 71 11120485 10.1016/s1368-8375(00)00059-2

[B137] Hotz B Arndt M Dullat S Bhargava S Buhr HJ Hotz HG Epithelial to mesenchymal transition: expression of the regulators snail, slug, and twist in pancreatic cancer Clin Cancer Res 2007 13 4769 4776 17699854 10.1158/1078-0432.CCR-06-2926

[B138] Jemal A Siegel R Ward E Hao Y Xu J Thun MJ Cancer statistics, 2009 CA Cancer J Clin 2009 59 225 249 19474385 10.3322/caac.20006

[B139] Roy H Smyrk T Koetsier J Victor T Wali R The transcriptional repressor SNAIL is overexpressed in human colon cancer Dig Dis Sci 2005 50 42 46 15712635 10.1007/s10620-005-1275-z

[B140] Fan F Samuel S Evans KW Lu J Xia L Zhou Y Sceusi E Tozzi F Ye XC Mani SA Ellis LM Overexpression of Snail induces epithelial-mesenchymal transition and a cancer stem cell-like phenotype in human colorectal cancer cells Cancer Med 2012 1 5 16 23342249 10.1002/cam4.4 PMC3544430

[B141] Yu Q Zhang K Wang X Liu X Zhang Z Expression of transcription factors snail, slug, and twist in human bladder carcinoma J Exp Clin Cancer Res 2010 29 119 20809941 10.1186/1756-9966-29-119 PMC2942802

[B142] Bruyere F Namdarian B Corcoran NM Pedersen J Ockrim J Voelzke BB Mete U Costello AJ Hovens CM Snail expression is an independent predictor of tumor recurrence in superficial bladder cancers Urol Oncol 2010 28 591 596 19162513 10.1016/j.urolonc.2008.11.005

[B143] Poser I Dominguez D Garcia de Herreros A Varnai A Buettner R Bosserhoff AK Loss of E-cadherin expression in melanoma cells involves up-regulation of the transcriptional repressor Snail J Biol Chem 2001 276 24661 24666 11323412 10.1074/jbc.M011224200

[B144] Kudo-Saito C Shirako H Takeuchi T Kawakami Y Cancer metastasis is accelerated through immunosuppression during Snail-induced EMT of cancer cells Cancer Cell 2009 15 195 206 19249678 10.1016/j.ccr.2009.01.023

[B145] Saito T Oda Y Tsuneyoshi M E-cadherin gene mutations frequently occur in synovial sarcoma as a determinant of histological features Am J Pathol 2001 159 2117 2124 11733362 10.1016/s0002-9440(10)63063-5 PMC1850581

[B146] Jemal A Bray F Center MM Ferlay J Ward E Forman D Global cancer statistics CA Cancer J Clin 2011 61 69 90 21296855 10.3322/caac.20107

[B147] Delahunt B Miller RJ Srigley JR Evans AJ Samaratunga H Gleason grading: past, present and future Histopathology 2012 60 75 86 22212079 10.1111/j.1365-2559.2011.04003.x

[B148] Pecina-Slaus N Tumor suppressor gene E-cadherin and its role in normal and malignant cells Cancer Cell Int 2003 3 17 18 14613514 10.1186/1475-2867-3-17 PMC270068

[B149] Edwards IJ Proteoglycans in prostate cancer Nat Rev Urol 2012 21 196 206 22349653 10.1038/nrurol.2012.19

[B150] Smith B Odero-Marah V The role of Snail in prostate cancer Cell Adh Migr 2012 6 433 441 23076049 10.4161/cam.21687 PMC3496681

[B151] Nackaerts K Verbeken E Deneffe G Vanderschueren B Demedts M David G Heparan sulfate proteoglycan expression in human lung-cancer cells Int J Cancer 1997 74 335 345 9221815 10.1002/(sici)1097-0215(19970620)74:3<335::aid-ijc18>3.0.co;2-a

[B152] Poblete C Fulla J Gallardo M Munoz V Castellon EA Gallegos I Contreras HR Increased SNAIL expression and low syndecan levels are associated with high Gleason grade in prostate cancer Int J Oncol 2014 44 647 654 24424718 10.3892/ijo.2014.2254 PMC3928469

[B153] Chen Z Li S Huang K Zhang Q Wang J Li X Hu T Wang S Yang R Jia Y Sun H Tang F Zhou H Shen J Ma D Wang S The nuclear protein expression levels of SNAI1 and ZEB1 are involved in the progression and lymph node metastasis of cervical cancer via the epithelial-mesenchymal transition pathway Hum Pathol 2013 44 2097 2105 23791009 10.1016/j.humpath.2013.04.001

[B154] Reya T Morrison SJ Clarke MF Weissman IL Stem cells, cancer, and cancer stem cells Nature 2001 414 105 111 11689955 10.1038/35102167

[B155] Al-Hajj M Wicha MS Benito-Hernandez A Morrison SJ Clarke MF Prospective identification of tumorigenic breast cancer cells Proc Natl Acad Sci U S A 2003 100 3983 3988 12629218 10.1073/pnas.0530291100 PMC153034

[B156] Jones RJ Matsui WH Smith BD Cancer stem cells: are we missing the target? J Natl Cancer Inst 2004 96 583 585 15100335 10.1093/jnci/djh095

[B157] Takahashi K Yamanaka S Induction of pluripotent stem cells from mouse embryonic and adult fibroblast cultures by defined factors Cell 2006 126 663 676 16904174 10.1016/j.cell.2006.07.024

[B158] Moon JH Heo JS Kim JS Jun EK Lee JH Kim A Kim J Kim J Whang KY Kang YK Yeo S Lim HJ Han DW Kim DW Oh S Yoon BS Schöler HR You S Reprogramming fibroblasts into induced pluripotent stem cells with Bmi1 Cell Res 2011 21 1305 1315 21709693 10.1038/cr.2011.107 PMC3193470

[B159] Moon JH Yun W Kim J Hyeon S Kang PJ Park G Kim A Oh S Whang KY Kim DW Yoon BS You S Reprogramming of mouse fibroblasts into induced pluripotent stem cells with Nanog Biochem Biophys Res Commun 2013 431 444 449 23333380 10.1016/j.bbrc.2012.12.149

[B160] Zhu L Qin H Li PY Xu SN Pang HF Zhao HZ Li DM Zhao Q Response gene to complement-32 enhances metastatic phenotype by mediating transforming growth factor beta-induced epithelial-mesenchymal transition in human pancreatic cancer cell line BxPC-3 J Exp Clin Cancer Res 2012 31 29 22458379 10.1186/1756-9966-31-29 PMC3337240

[B161] Huang J Song H Liu B Yu B Wang R Chen L Expression of Notch-1 and its clinical significance in different histological subtypes of human lung adenocarcinoma J Exp Clin Cancer Res 2013 32 84 24423157 10.1186/1756-9966-32-84 PMC3818984

[B162] Fujii R Imanishi Y Shibata K Sakai N Sakamoto K Shigetomi S Habu N Otsuka K Sato Y Watanabe Y Ozawa H Tomita T Kameyama K Fujii M Ogawa K Restoration of E-cadherin expression by selective Cox-2 inhibition and the clinical relevance of the epithelial-to-mesenchymal transition in head and neck squamous cell carcinoma J Exp Clin Cancer Res 2014 33 40 24887090 10.1186/1756-9966-33-40 PMC4030015

[B163] Zhuo W Wang Y Zhuo X Zhang Y Ao X Chen Z Knockdown of Snail, a novel zinc finger transcription factor, via RNA interference increases A549 cell sensitivity to cisplatin via JNK/mitochondrial pathway Lung Cancer 2008 62 8 14 18372076 10.1016/j.lungcan.2008.02.007

[B164] Hsu DS Lan HY Huang CH Tai SK Chang SY Tsai TL Chang CC Tzeng CH Wu KJ Kao JY Yang MH Regulation of excision repair cross-complementation group 1 by Snail contributes to cisplatin resistance in head and neck cancer Clin Cancer Res 2010 16 4561 4571 20823140 10.1158/1078-0432.CCR-10-0593

[B165] Haslehurst AM Koti M Dharsee M Nuin P Evans K Geraci J Childs T Chen J Li J Weberpals J Davey S Squire J Park PC Feilotter H EMT transcription factors snail and slug directly contribute to cisplatin resistance in ovarian cancer BMC Cancer 2012 12 91 22429801 10.1186/1471-2407-12-91 PMC3342883

[B166] Kurrey NK Jalgaonkar SP Joglekar AV Ghanate AD Chaskar PD Doiphode RY Bapat SA Snail and slug mediate radioresistance and chemoresistance by antagonizing p53-mediated apoptosis and acquiring a stem-like phenotype in ovarian cancer cells Stem Cells 2009 27 2059 2068 19544473 10.1002/stem.154

[B167] Yin T Wang C Liu T Zhao G Zha Y Yang M Expression of Snail in pancreatic cancer promotes metastasis and chemoresistance J Surg Res 2007 141 196 203 17583745 10.1016/j.jss.2006.09.027

[B168] Vega S Morales AV Ocana OH Valdes F Fabregat I Nieto MA Snail blocks the cell cycle and confers resistance to cell death Genes Dev 2004 18 1131 1141 15155580 10.1101/gad.294104 PMC415638

[B169] Baritaki S Yeung K Palladino M Berenson J Bonavida B Pivotal roles of snail inhibition and RKIP induction by the proteasome inhibitor NPI-0052 in tumor cell chemoimmunosensitization Cancer Res 2009 69 8376 8385 19843864 10.1158/0008-5472.CAN-09-1069

[B170] Jazirehi AR Huerta-Yepez S Cheng G Bonavida B Rituximab (chimeric anti-CD20 monoclonal antibody) inhibits the constitutive nuclear factor-{kappa}B signaling pathway in non-Hodgkin's lymphoma B-cell lines: role in sensitization to chemotherapeutic drug-induced apoptosis Cancer Res 2005 65 264 276 15665303

[B171] Vega MI Baritaki S Huerta-Yepez S Martinez-Paniagua MA Bonavida B A potential mechanism of rituximab-induced inhibition of tumorgrowth through its sensitization to tumor necrosis factor-related apoptosis-inducing ligand-expressing host cytotoxic cells Leuk Lymphoma 2011 52 108 121 21133714 10.3109/10428194.2010.531408

[B172] Akalay I Janji B Hasmim M Noman MZ Thiery JP Mami-Chouaib F Chouaib S EMT impairs breast carcinoma cell susceptibility to CTL-mediated lysis through autophagy induction Autophagy 2013 9 1104 1106 23635487 10.4161/auto.24728 PMC3722321

[B173] Akalay I Janji B Hasmim M Noman MZ André F De Cremoux P Bertheau P Badoual C Vielh P Larsen AK Sabbah M Tan TZ Keira JH Hung NT Thiery JP Mami-Chouaib F Chouaib S Epithelial-to mesenchymal transition and autophagy induction in breast carcinoma promote escape from T-cell-mediated lysis Cancer Res 2013 73 2418 2427 23436798 10.1158/0008-5472.CAN-12-2432

[B174] Lee SH Lee SJ Chung JY Jung YS Choi SY Hwang SH Choi D Ha NC Park BJ p53, secreted by K-Ras-Snail pathway, is endocytosed by K-Ras-mutated cells; implication of target-specific drug delivery and early diagnostic marker Oncogene 2009 28 2005 2014 19347028 10.1038/onc.2009.67

[B175] Lee SH Shen GN Jung YS Lee SJ Chung JY Kim HS Xu Y Choi Y Lee JW Ha NC Song GY Park BJ Antitumor effect of novel small chemical inhibitors of Snail-p53 binding in K-Ras-mutated cancer cells Oncogene 2010 29 4576 4587 20531295 10.1038/onc.2010.208

[B176] Harney A Meade T LaBonne C Targeted inactivation of snail family EMT regulatory factors by a Co(III)-Ebox conjugate PLoS One 2012 7 e32318 22393397 10.1371/journal.pone.0032318 PMC3290632

[B177] Javaid S Zhang J Anderssen E Black JC Wittner BS Tajima K Ting DT Smolen GA Zubrowski M Desai R Maheswaran S Ramaswamy S Whetstine JR Haber DA Dynamic chromatin modification sustains epithelial-mesenchymal transition following inducible expression of Snail-1 Cell Rep 2013 5 1679 1689 24360956 10.1016/j.celrep.2013.11.034 PMC4034764

[B178] Shah P Gau Y Sabnis G Histone deacetylase inhibitor entinostat reverses epithelial to mesenchymal transition of breast cancer cells by reversing the repression of E-cadherin Breast Cancer Res Treat 2014 143 99 111 24305977 10.1007/s10549-013-2784-7

[B179] Hatzivassiliou G Haling JF Chen H Song K Price S Heald R Hewitt JF Zak M Peck A Orr C Merchant M Hoeflich KP Chan J Luoh SM Anderson DJ Ludlam MJ Wiesmann C Ultsch M Friedman LS Malek S Belvin M Mechanism of MEK inhibition determines efficacy in mutant KRAS- versus BRAF-driven cancers Nature 2013 501 232 236 23934108 10.1038/nature12441

[B180] Miller C Oliver K Farley J MEK1/2 inhibitors in the treatment of gynecologic malignancies Gynecol Oncol 2014 133 128 137 24434059 10.1016/j.ygyno.2014.01.008

[B181] McCubrey JA Steelman LS Chappell WH Abrams SL Franklin RA Montalto G Cervello M Libra M Candido S Malaponte G Mazzarino MC Fagone P Nicoletti F Bäsecke J Mijatovic S Maksimovic-Ivanic D Milella M Tafuri A Chiarini F Evangelisti C Cocco L Martelli AM Ras/Raf/MEK/ERK and PI3K/PTEN/Akt/mTOR cascade inhibitors: how mutations can result in therapy resistance and how to overcome resistance Oncotarget 2012 3 1068 1111 23085539 10.18632/oncotarget.659 PMC3717945

[B182] http://clinicaltrials.gov *NIH Database.*. .

[B183] Mimasu S Sengoku T Fukuzawa S Umehara T Yokoyama S Crystal structure of histone demethylase LSD1 and tranylcypromine at 2.25 Å Biochem Biophys Res Commun 2008 366 15 22 18039463 10.1016/j.bbrc.2007.11.066

[B184] http://pubchem.ncbi.nlm.nih.gov/summary/summary.cgi?cid=444732&loc=ec_rcs *Pubchem Database.*. []

[B185] http://pubchem.ncbi.nlm.nih.gov/summary/summary.cgi?cid=4688&loc=ec_rcs *Pubchem Database.*. []

[B186] http://pubchem.ncbi.nlm.nih.gov/summary/summary.cgi?cid=6918837 *Pubchem Database.*. []

[B187] http://pubchem.ncbi.nlm.nih.gov/summary/summary.cgi?cid=4261 *Pubchem Database.*. []

